# Supplementary material for: Preclinical Evaluation of ADVM-022, a Novel Gene Therapy Approach to Treating Wet Age-Related Macular Degeneration
Source: Mol Ther. 2018 Nov 13;27(1):118–29. doi: 10.1016/j.ymthe.2018.11.003 (PMC6319194; doi:10.1016/j.ymthe.2018.11.003)
Supplement: Document S1. Supplemental Materials and Methods, Table S1, and Figures S1–S5 [file mmc1.pdf]

YMTHE, Volume *27*

Supplemental Information

Preclinical Evaluation of ADVM-022, a Novel Gene Therapy Approach to Treating Wet Age-Related Macular Degeneration

Ruslan Grishanin, Brian Vuillemenot, Pallavi Sharma, Annahita Keravala, Judith Greengard, Claire Gelfman, Mark Blumenkrantz, Matthew Lawrence, Wenzheng Hu, Szilárd Kiss, and Mehdi Gasmi

SUPPLEMENTAL INFORMATION

Supplemental Materials and Methods

AAV2.7m8-CMV-GFP Vector Description

AAV2.7m8-CMV-GFP utilizes the AAV2.7m8 capsid, a variant of AAV2 that includes a 10-amino acid insertion in Loop IV of the AAV2 viral capsid proteins (VP1-3). The DNA genome consists of the viral inverted terminal repeats (ITRs) from AAV2 flanking the expression cassette, CMV-GFP. This cassette includes the human cytomegalovirus (CMV) immediate early enhancer and promoter driving expression of enhanced green fluorescent protein (eGFP) followed by an SV40 poly adenylation (poly A) signal. The cassette also contains a synthetic intron 5` to the eGFP cDNA.

Expression following transuction of pig retinal explants with AAV.7m8-CMV-sFLT1 or AAV.7m8-C11-CO.sFLT

Pig neuroretina explants (n=3), placed flat in transwell inserts with the photoreceptor layer facing down, were transduced with either AAV.7m8-CMV-sFLT1 or AAV.7m8-C11-CO.sFLT at a multiplicity of infection (moi) of $2x10^4$. Explants were cultured in Neurobasal A media supplemented with B27 and L-Glutamine for 2 weeks with media changes every 2-3 days. Expression of secreted sFlt1 protein was measured in the 3 day-old culture medium collected at day 14 post-transduction. Concentration of secreted sFLT1 protein was analyzed using the Human VEGF R1/Flt-1 DuoSet ELISA from R&D systems.

cSLO imaging and immunofluorescence following AAV2.7m8-CMG-GFP vector transduction

The transduction efficiency in the African green monkey retina was evaluated by immunofluorescence 12 weeks post IVT injection of AAV2.7m8-CMV-GFP ($5x10^{11}$ vg/eye). The eyes were enucleated immediately after euthanasia and fixed by immersion in 4% paraformaldehyde for 24 hours at 4 ºC, and then transferred to 30% sucrose in phosphate buffer saline (PBS) followed by storage at 4 ºC. The retina was separated from the sclera and flatmounts were prepared by making 4 radial cuts at the periphery and covered with a coverslip using 30% sucrose solution in PBS. GFP fluorescence from the retinal flatmounts was imaged at 5X magnification upon excitation at 488nm; tiled images were collected and stitched together using Zeiss software (Carl Zeiss ZEN 2 Blue Edition).

Immunohistochemistry was performed on histological sections containing retina and RPE prepared by embedding tissue in frozen OCT, and sectioned at 7µm thickness prior to mounting on charged slides (VWR). Prior to incubation with the appropriate antibody, tissue sections were blocked for 1 hour at room temperature with 5% normal donkey serum (Jackson ImmunoResearch) and 6% bovine serum albumin (Amresco) in PBS (Gibco).

GFP was detected from the AAV2.7m8-CMV-GFP-trasduced cells using chicken polyclonal GFP antibody (Abcam) at 10µg/mL, followed by incubation with donkey anti-chicken IgY Alexa Flour 488 conjugated secondary antibody (Jackson ImmunoResearch) at 5µg/mL. To identify cone photoreceptors, rhodamine-conjugated peanut agglutinin (Vector Labs) was used at 5µg/mL. Retinal ganglion cells were stained with monoclonal anti-beta III tubulin (TUJ1) antibody (Abcam), at 5 µg/ml, and detected using donkey anti-mouse antibody conjugated with Alexa 647 (Thermo Scientific) at 5 µg/ml. Rod photoreceptors were stained using anti-rhodopsin 4D2 monoclonal antibody (Millipore) at 0.4µg/mL and detected with secondary donkey-anti-mouse antibody conjugated with Alexa Fluor 555 (ThermoFisher) at 5µg/mL. Nuclei were stained with DAPI Nucleic Acid Stain (Invitrogen), used at 0.5µM. Stained retinal sections were covered with a coverslip with mounting medium applied.

Supplemental Table 1: Summary of eyes/lesions excluded from scoring

Treatment	Animal	Number of lesions applied		Excluded eyes/ lesions	
		OD	OS	OD	OS
ADVM-022	A014	9	9		
	A255	9	9		
	A079	8	9	Eye excluded at post-laser week 2 and 4	Eye excluded at week 2 post-laser
	A066	9	9		
Vehicle	A260	9	9	Eye excluded at post-laser week 2	
	K973	9	9		
	A090	9	9		
	A191	9	9		
Aflibercept	A386	9	9		
	A540	9	9		Eye excluded at week 2 post-laser
	A678	9	9		Eye excluded from the scoring at week 2 post-laser
	A681	9	6	Eye excluded from the scoring at week 2 post-laser	Eye excluded from the scoring at week 2 post-laser; 2 lesions excluded at week 4 post-laser

Supplemental Figures

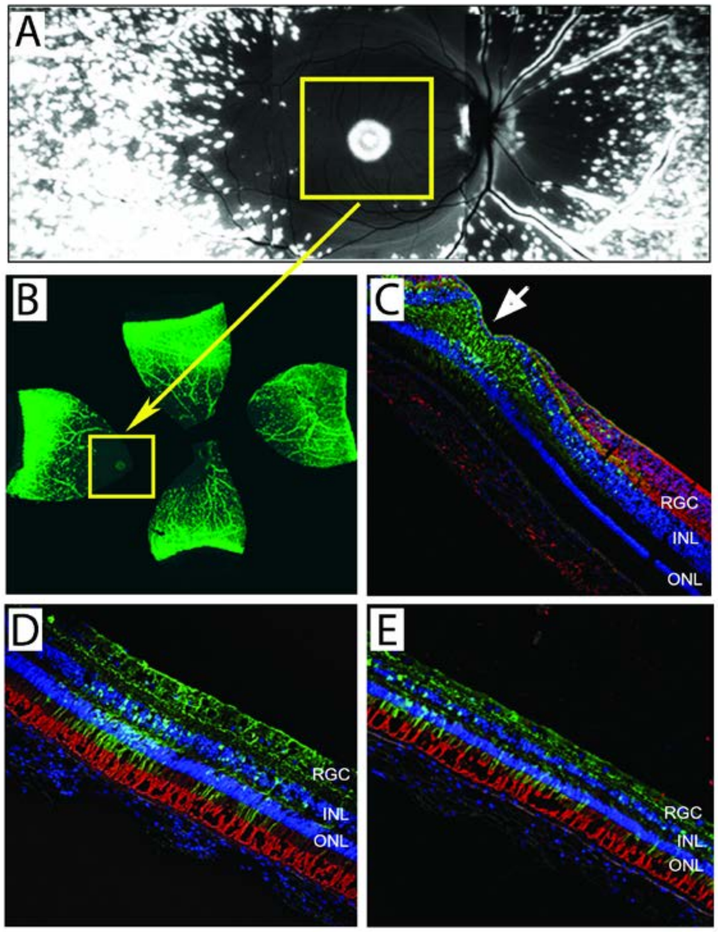

Figure S1. AAV2.7m8 driven GFP expression in the retina of African green monkey. AAV2.7m8-CMV-GFP (5x10^{11} vg/eye) was injected IVT, and retinas were analyzed for the distribution of GFP expression 12 weeks post-injection.

A. Expression of GFP in the fundus of African green monkey imaged by confocal scanning laser ophthalmoscopy (cSLO). The box indicates GFP expression in the fovea. **B.** Retinal flat mount demonstrating GFP transgene expression in fovea, at the mid-periphery out to ora serrata. **C**. Retinal section through fovea and perifoveal area. White arrow indicates foveal pit. GFP is seen in cones, outer plexiform layer, sparse cells in inner nuclear layer (INL) and sparse retinal ganglion cells (RGC). (Red: TUJ-1, retinal ganglion cells). **D**. In mid-periphery, GFP expression is seen within the INL, inner plexiform layer of the retina, RGC layer and in patches of rod photoreceptors. (Red: rhodopsin, rod photoreceptors) **E.** In the periphery, GFP expression can be seen within the photoreceptor layer and the outer nuclear layer (ONL). (Red: rhodopsin). In all images green identifies GFP transgene expressing cells, and DAPI staining was used to identify cell nuclei. The disruptions in the outer segment region (D and E) are artifacts in frozen section preparation.

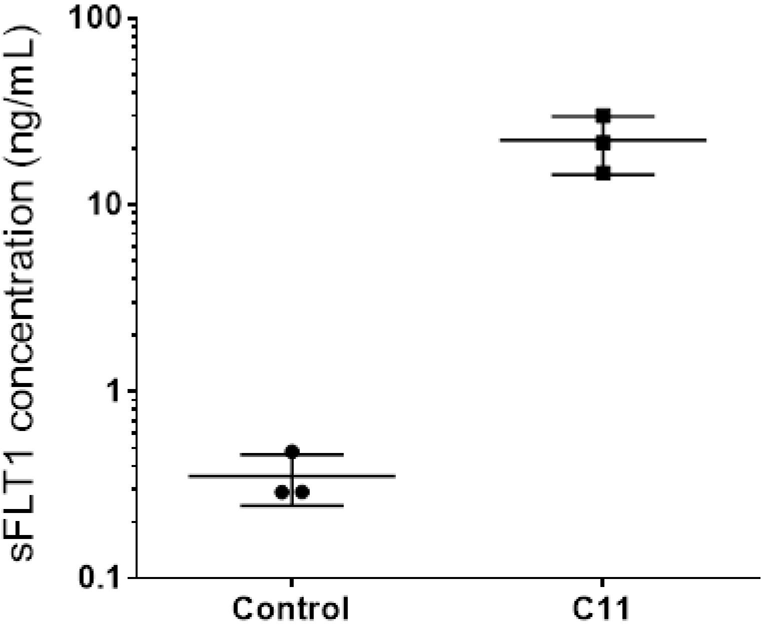

Figure S2. Expression of recombinant protein in pig retinal explants transduced with AAV2.7m8 vector with the original sFlt1-expression cassette under control of CMV early enhancer/promoter and SV40 poly-adenylation signal compared with AAV2.7m8 with codon-optimized Flt1 under control of C11 combination of regulatory elements. Human Flt1 receptor ectodomain sFlt1 was used as a reporter in the optimization screens. Control: AAV2.7m8CMV-sFlt1 (CMV early enhancer/promoter, followed by sFlt1 sequence with SV40 polyA signal), C11: AAV.7m8-C11-CO.sFlt1. Mean values ± SEM are indicated.

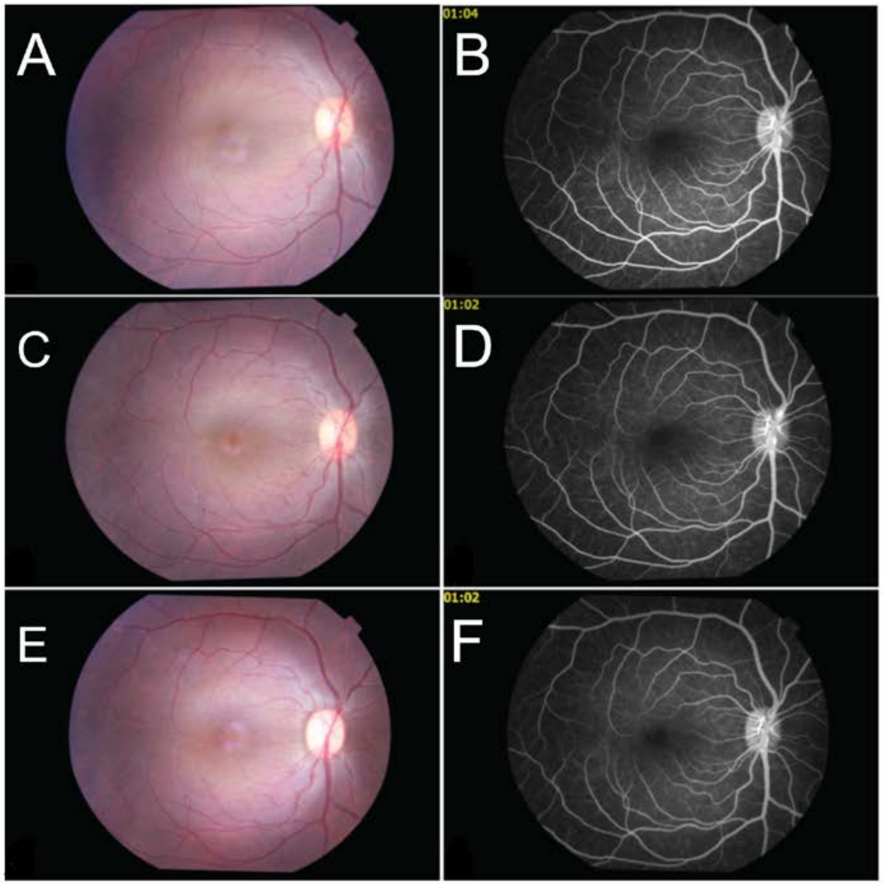

Figure S3: IVT administration of ADVM-022 does not result in changes in retinal morphology, vascular integrity or optic nerve head. Representative color fundus photographs and early phase fluorescence angiograms of eyes receiving ADVM-022 from baseline to 12.5 months. Photographs were obtained from the eye A255 OD at baseline (**A** and **B**), 6 months (**C** and **D**), and 12.5 months (**E** and **F**).

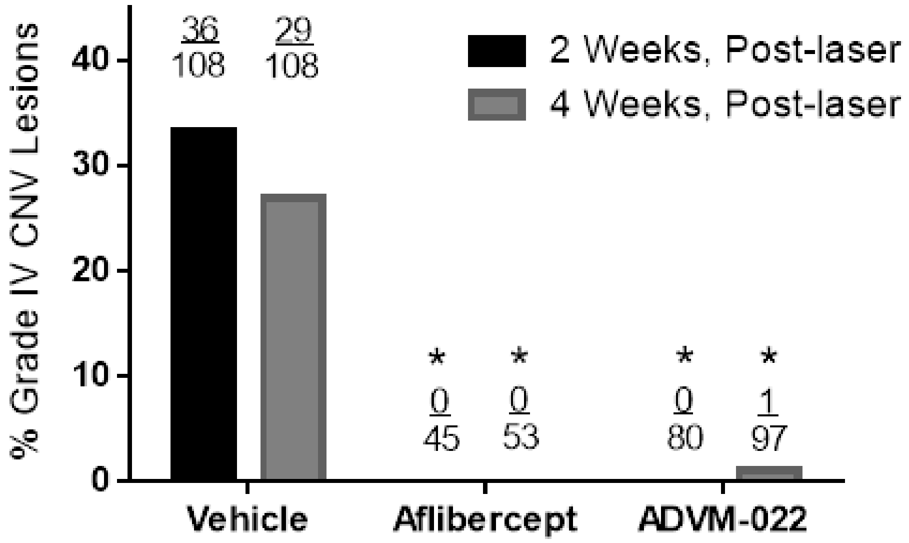

Figure S4. Single dose IVT ADVM-022 significantly reduces the incidence of Grade IV lesions when administered 56 days prior to laser-induced CNV. 6 animals received bilateral IVT injections of ADVM-022 (2×10^{12} vg/eye), and 6 animals of control group received IVT vehicle injections. Laser induction of CNV was performed 56 days after dosing. Standard of care aflibercept was used as a positive control. It was injected immediately after the laser procedure, to the eyes of 3 previously treatment-naïve animals. The Grade IV lesion incidence was scored 2 and 4 days after the CNV induction. The decrease in Grade IV lesions between the ADVM-022 and aflibercept groups compared with the control group was statistically significant ($p < 0.0001$). The incidence of Grade IV lesions was similar between the ADVM-022 and aflibercept groups.

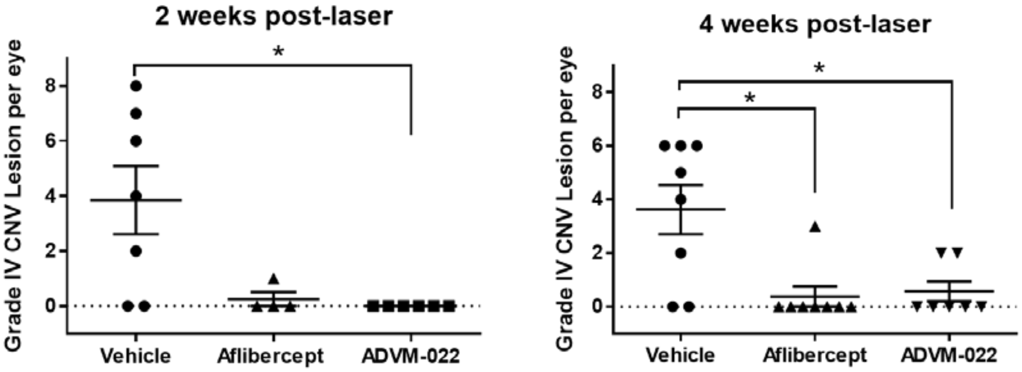

Figure S5. Single dose IVT ADVM-022 reduces incidence of grade IV lesions per individual eye, in the laser-induced CNV African green monkey model, 13 months after the therapy delivery. To address the potential eye-to-eye variability in response to the treatments, the treatment groups were also compared based on the counts of Grade IV lesions per eye, using the Mann-Whitney U-test. This analysis confirmed a statistically significant lower incidence of Grade IV lesions in the eyes treated with ADVM-022 compared with vehicle 2 weeks and 4 weeks post laser. The effect of aflibercept did not reach statistical significance at 2 weeks due to the low number of lesions observable for scoring, and it was statistically significant at 4 weeks post laser. There was no significant difference between ADVM-022 and aflibercept at either time point. At 2 weeks post-laser, 7 vehicle-injected eyes, 4 aflibercept injected eyes and 6 ADVM-022 injected eyes were suitable for scoring; at 4 weeks, 8 vehicle treated eyes, 8 aflibercept-injected eyes and 7 ADVM-022-injected eyes were scored by the treatment-masked investigator. Means with SEM are indicated. * $P < 0.05$.
