# Supplementary material for: Preclinical Evaluation of ADVM-022, a Novel Gene Therapy Approach to Treating Wet Age-Related Macular Degeneration
Source: Mol Ther. 2018 Nov 13;27(1):118–29. doi: 10.1016/j.ymthe.2018.11.003 (PMC6319194; doi:10.1016/j.ymthe.2018.11.003)
Supplement: Document S2. Article plus Supplemental Information [file mmc2.pdf]

Molecular Therapy
Original Article

Preclinical Evaluation of ADVM-022, a Novel Gene Therapy Approach to Treating Wet Age-Related Macular Degeneration

Ruslan Grishanin,[1] Brian Vuillemenot,[1] Pallavi Sharma,[1] Annahita Keravala,[1] Judith Greengard,[1] Claire Gelfman,[1] Mark Blumenkrantz,[2] Matthew Lawrence,[3] Wenzheng Hu,[3] Szilárd Kiss,[4] and Mehdi Gasmi[1]

[1]Adverum Biotechnologies, Menlo Park, CA, USA; [2]Stanford University, Palo Alto, CA, USA; [3]RxGen Inc., New Haven, CT, USA; [4]Weill Cornell Medical College, New York, NY, USA

Inhibition of vascular endothelial growth factor, a key contributor to the choroidal neovascularization associated with wet age-related macular degeneration, is the mode of action of several approved therapies, including aflibercept, which requires frequent intravitreal injections to provide clinical benefit. Lack of compliance with the dosing schedule may result in recurrence of active wet macular degeneration, leading to irreversible vision impairment. Gene therapy providing sustained anti-vascular endothelial growth factor levels in the retina following a single injection could drastically reduce the treatment burden and improve visual outcomes. ADVM-022, an adeno-associated virus vector encoding aflibercept, is optimized for intravitreal delivery and strong protein expression. Here, we report the long-term expression and efficacy of ADVM-022-derived aflibercept in a laser-induced choroidal neovascularization model in non-human primates. Intravitreal administration of ADVM-022 was well tolerated and resulted in sustained aflibercept levels. In addition, ADVM-022 administration 13 months before lasering prevented the occurrence of clinically relevant choroidal neovascularization lesions, similar to animals that received a bolus of intravitreal aflibercept (standard of care) at the time of lesioning. These results demonstrate that a single intravitreal administration of ADVM-022 may provide a safe and effective long-term treatment option for wet macular degeneration and may ultimately improve patients' visual outcomes.

INTRODUCTION

Age-related macular degeneration (AMD) is the most common cause of vision impairment in individuals 50 years of age and older. Degeneration of the macula, which plays a critical role in detailed central vision, leads to blurred or distorted areas within the central visual field.[1,2] Approximately 10% to 20% of patients with AMD develop abnormal blood vessel formation in the choroid layer area under the macula. This choroidal neovascularization (CNV) results in the "wet" or exudative form of AMD (wAMD), which is characterized by leakage of blood and fluid from the abnormal new vessels into the retina. Accumulation of fluid within the retina leads to photoreceptor degeneration, secondary scarring,[2] and ultimately vision loss. Although the wet form accounts for a minority of all cases of AMD, it accounts for 90% of AMD-related vision loss.[2]

Vascular endothelial growth factor (VEGFA) plays a key role in the development of CNV and its sequelae in wAMD.[3] As such, it is a key target for therapeutic intervention in the treatment of the disease. Three recombinant anti-VEGFA protein therapies (ranibizumab,[4] bevacizumab,[5] and aflibercept[6]) that block VEGFA-induced neovascularization have revolutionized the treatment of wAMD and have become standard of care.

Despite the efficacy of protein-based anti-VEGFA therapies, a key drawback to their use is the need for chronic intravitreal (IVT) injections every 4 to 8 weeks that involve frequent office visits, injection discomfort, and logistical burden placed on relatives and caretakers in addition to costs of treatment.[7] As a result, long-term follow-up of wAMD has shown that mean dosing frequencies are less than half the recommended injections, which leads to disease progression and vision loss.[8–10] Conversely, patients receiving regular and more frequent anti-VEGFA therapy show significantly improved vision.[11] In addition to these compliance issues, monthly administration results in uneven exposure, with peak and trough levels of the anti-VEGFA protein throughout the dosing cycle.[12]

Gene-based delivery of anti-VEGFA proteins may provide a promising alternative to protein-based therapies. This approach can, potentially, generate robust and sustained levels of therapeutic proteins in the outer retina and choroid where CNV occurs and potentially decrease the need for frequent injections. Several anti-VEGFA gene-therapy approaches have been tried in the past using a variety of vector systems and different anti-VEGFA transgenes.[13–17] Unfortunately, these efforts led to limited efficacy in the clinic, likely due to a combination of issues related to the route of administration

Received 15 June 2018; accepted 7 November 2018;
https://doi.org/10.1016/j.ymthe.2018.11.003.

Correspondence: Mehdi Gasmi, Adverum Biotechnologies, 1035 O'Brien Dr., Menlo Park, CA 94025, USA.
E-mail: mehdi@adverum.com

 © 2018 ADVERUM BIOTECHNOLOGIES, INC.
This is an open access article under the CC BY license (http://creativecommons.org/licenses/by/4.0/).

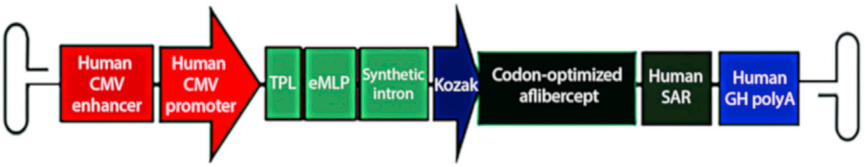

Figure 1. Design of the Aflibercept Expression Cassette (C11)

The aflibercept transgene expression cassette is flanked by AAV2 inverted terminal repeats (ITRs). The C11.CO.aflibercept cassette includes regulatory elements including the human cytomegalovirus (CMV) immediate-early enhancer and promoter, an adenovirus tripartite leader sequence (TPL) followed by an enhancer element from the major late promoter (eMLP), a synthetic intron, and a Kozak sequence driving expression of aflibercept. The cDNA of aflibercept is followed by a human scaffold attachment region (SAR) and the human growth hormone (GH) polyadenylation site. Aflibercept is a recombinant chimeric protein consisting of the vascular endothelial growth factor (VEGFA) binding portion of human VEGFR-1 (domain 2) and VEGFR-2 (domain 3 or KDR) fused to the Fc portion of human IgG1 immunoglobulin.

(subretinal versus IVT), vector transduction efficiency, nature of anti-VEGFA transgenes, and vector dose.

ADVM-022, a novel recombinant adeno-associated virus (AAV)-based therapy for wAMD, is optimized for IVT administration and robust protein expression, circumventing some of the limitations of other AAV vectors used in the past. ADVM-022 utilizes the AAV2.7m8 capsid, which has been engineered from AAV2 by directed evolution and screened for highly efficient retinal transduction following IVT administration[18] and which carries a strong, ubiquitous expression cassette encoding a codon-optimized cDNA of the aflibercept protein, a validated therapy for wAMD. Delivery of ADVM-022 therefore has the potential to treat neovascularization that precedes vision loss.

Herein, we report the long-term effect of ADVM-022 more than 1 year post-IVT administration in the laser-induced CNV model in non-human primates (NHPs). ADVM-022 was well-tolerated with no serious adverse safety-related findings, with observations limited to mild, self-resolving inflammation and no changes to macular thickness or volume. In addition, more than a year post-vector-administration, ADVM-022 provided robust aflibercept expression and was highly effective at preventing the occurrence of clinically relevant grade IV laser-induced CNV lesions, similar to a bolus of aflibercept recombinant protein administered at the time of lesioning.

RESULTS

Vector Characterization

ADVM-022 (AAV2.7m8-C11.CO.aflibercept) utilizes a novel variant of the AAVserotype 2 (AAV2) capsid as a vector for delivering and encoding aflibercept, a recombinant chimeric protein comprising the VEGFA-binding portions of the extracellular domains of human VEGFA receptors 1 and 2 and the fragment crystallizable region (Fc) portion of human immunoglobulin (IgG1).[19,20] ADVM-022 was produced in the baculovirus expression system in Sf9 cells where two baculoviruses were used, one encoding the genes for AAV2 Rep and AAV2.7m8 Cap proteins, and the other encoding the vector genome carrying the codon-optimized aflibercept cDNA expression cassette. ADVM-022 was purified by chromatography and filtration steps allowing separation from cell and baculovirus contaminants as well as the enrichment of the purified product in full capsids.

AAV2.7m8 was discovered by directed evolution[18] and is a variant of AAV2, which includes a 10-amino-acid insertion in loop IV of the AAV2 viral structural proteins (VP1-3). It has an improved transduction efficiency *in vitro* and *in vivo* when administered IVT, compared with AAV2 when tested in rodents, non-human primates, and human retinal explants.[18,21] We tested this vector variant for the efficacy of gene delivery to the retina in the African green monkey, following IVT injection of AAV2.7m8-GFP. The tropism of the AAV2.7m8 capsid and distribution of GFP-transduced cells in African green monkeys, observed by fundus fluorescence imaging by cSLO (confocal scanning laser ophthalmoscopy), corroborated the data described in Dalkara et al.,[18] with strong transduction in fovea and peripheral retina, areas known to have a thin inner limiting membrane (Figure S1). The ADVM-022 expression cassette consisted of a codon-optimized aflibercept cDNA and a combination of regulatory elements to enhance protein expression (Figure 1). This combination, designated C11, was generated by random association of different regulatory elements, including the CMV promoter, and identified by screening in various cell lines and pig retinal explants to evaluate their efficiency in retinal cells. Figure S2 shows an example of enhanced expression of the recombinant protein sFlt1 in porcine retinal explants transduced with AAV2.7m8 vector carrying the improved expression cassette, C11, compared with AAV2.7m8 carrying a cassette under control of the CMV early enhancer-promoter.

Aflibercept Expression in Vitreous Humor from ADVM-022

The capacity of ADVM-022 to deliver persistent and pharmacologically relevant levels of aflibercept following IVT injection was evaluated in NHPs. Seven adult monkeys received bilateral 50 μL IVT injections of $\sim 2 \times 10^{12}$ viral genomes (vg) of ADVM-022 per eye (Table 1). Although higher than what is commonly used in IVT studies (generally in the 10^{10}–10^{11} range),[21] previous pilot studies with AAV2.7m8 vectors had shown that this vector was well tolerated at this dose. Another seven monkeys received 50-μL bilateral injections of formulation buffer (vehicle group).

To assess expression levels, vitreous humor was sampled according to the schedule presented in Table 2 and analyzed by an ELISA specific for free (unbound) aflibercept. No aflibercept protein was detected in the vitreous humor of the vehicle control group. In the ADVM-022-treated animals, vitreous aflibercept levels were robust and remained elevated in all animals between 3 and 9 months (averaging 3.5 ±

Table 1. Study Design

Animal	Group	Subgroup[a]	Test Article	Injection Day	Dose/Volume per Eye[b]	Laser Treatment Month, Post-ADVM-022 IVT Dose
A014	1	1a	ADVM-022	day 0	2×10^{12} vg/50 μL	13
A066	1	1a	ADVM-022	day 0	2×10^{12} vg/50 μL	13
A079	1	1a	ADVM-022	day 0	2×10^{12} vg/50 μL	13
A255	1	1a	ADVM-022	day 0	2×10^{12} vg/50 μL	13
A055	1	1b	ADVM-022	day 0	2×10^{12} vg/50 μL	not lasered
A070	1	1b	ADVM-022	day 0	2×10^{12} vg/50 μL	not lasered
A075	1	1b	ADVM-022	day 0	2×10^{12} vg/50 μL	not lasered
A090	2	2a	vehicle	day 0	50 μL	13
A191	2	2a	vehicle	day 0	50 μL	13
A260	2	2a	vehicle	day 0	50 μL	13
K973	2	2a	vehicle	day 0	50 μL	13
A118	2	2b	vehicle	day 0	50 μL	not lasered
A194	2	2b	vehicle	day 0	50 μL	not lasered
K938	2	2b	vehicle	day 0	50 μL	not lasered
A386	3	3a	aflibercept	month 13[c]	1.2 mg/30 μL	13
A540	3	3a	aflibercept	month 13[c]	1.2 mg/30 μL	13
A678	3	3a	aflibercept	month 13[c]	1.2 mg/30 μL	13
A681	3	3a	aflibercept	month 13[c]	1.2 mg/30 μL	13

[a]At 12.5 months, group 1 and 2 animals were divided into subgroup a and b, respectively.
[b]All articles were delivered via intravitreal injection to both eyes.
[c]Injected immediately following laser photocoagulation procedure.

1.9 μg/mL across all animals and time points), albeit a slight decrease between months 7 and 9 in all animals, likely reflecting the variability of the assay (Figure 2A). One animal (A255) had very high expression in both eyes compared to the rest of the ADVM-022-treated animals, with average expression levels measuring 7.5 ± 0.95 μg/mL throughout the time course. Interestingly, the right eye of one animal (animal A055) showed a progressive decline (from 2.2 μg/mL at 3 months to 0.87 μg/mL at 16 months) in vitreous aflibercept levels over time while the protein levels in the left eye remained relatively stable throughout the study. Laser photocoagulation procedure did not affect levels of aflibercept protein in ADVM-022-treated animals; the levels of aflibercept detected before the laser procedure (9 months, 3.4 ± 2.0 μg/mL) and after the procedure (15.5 months, 3.4 ± 2.4 μg/mL) were indistinguishable ($p = 0.94$, paired t test).

Robust levels of aflibercept were also measured in retina and choroid tissues—the site of neovascularization in wAMD—in all animals, consistent with levels found in the vitreous (Figure 2B). Aflibercept levels were detected at lower levels in the aqueous humor after administration of ADVM-022 at 2×10^{12} vg/eye (e.g., 0.7 ± 0.2 μg/mL in aqueous humor, compared to 3.4 ± 0.5 μg/mL; mean ± SEM in vitreous humor) 7 months post-dose (data not shown). The aflibercept levels measured in aqueous humor of ADVM-022-treated eyes were comparable to levels described by Niwa et al.[22] from the aqueous humor of cynomolgus monkey eyes measured 2 weeks after IVT injection of 2 mg aflibercept.

Safety and Tolerability of ADVM-022

Routine clinical observations including body weight and food consumption were consistent with IVT administration of $\sim 2 \times 10^{12}$ vg/eye of ADVM-022 being well tolerated. There were no clinical signs indicative of ADVM-022 related systemic effects.

To evaluate the ocular safety and tolerability of ADVM-022 following IVT administration, eyes were assessed by slit-lamp biomicroscopy and fundoscopy prior to dosing and at different time points up to 12.5 months post-dose (shortly before laser treatment) in all seven animals in the ADVM-022 and vehicle control groups. In the vehicle control-treated animals evaluated, the ophthalmic exam findings were limited to fibrin strands occurring in 4 of 14 eyes and aqueous cells in 1 of 14 eyes during the 12.5 months of observation period prior to laser treatment. Ophthalmic effects, including aqueous cell infiltrates (mild to moderate), vitreous cell infiltrates (mild to moderate), keratic precipitates (mild to moderate), and incidental lens capsule deposits (mild to moderate), were observed in animals treated with ADVM-022 (Figure 3). The aqueous cell response peaked at 1 month and resolved by 3 months post-injection (Figure 3A) without anti-inflammatory treatment. In addition, fine white cell and some pigmented keratic precipitates were observed early in the study (up to 6 months post-dose), with the increasing occurrence of pigmented keratic precipitates at the later time points. (9–12.5 months). Keratic precipitate severity scores were mild to moderate and persisted for the duration of the study period and were not deemed clinically

Table 2. Vitreous Humor Sample Collection Schedule

Animal	Test Article	Baseline	3[a]	7[a]	9[a]	13[a]	15.5[a]	16[a]
A014	ADVM-022	+	+	+	+	–[b]	+	x
A066	ADVM-022	+	+	+	+	–[b]	+	x
A079	ADVM-022	+	+	+	+	–[b]	+	x
A255	ADVM-022	+	+	+	+	–[b]	+	x
A055	ADVM-022	+	+	+	+	+	–	+
A070	ADVM-022	+	+	+	+	+	–	+
A075	ADVM-022	+	+	+	+	+	–	+
A090	vehicle	+	+	+	+	–[b]	+	x
A191	vehicle	+	+	+	+	–[b]	+	x
A260	vehicle	+	+	+	+	–[b]	+	x
K973	vehicle	+	+	+	+	–[b]	+	x
A118	vehicle	+	+	+	+	+	–	+
A194	vehicle	+	+	+	+	+	–	+
K938	vehicle	+	+	+	+	+	–	+

+, Vitreous humor collected; –, vitreous humor not collected; x, vitreous humor collected at termination.
[a]Months following IVT delivery of test article or vehicle.
[b]Laser procedure administered; vitreous humor not collected.

significant by the facility veterinarian and ophthalmologist (Figure 3C). Vitreous cell response in ADVM-022-treated animals ranged from absent to moderate, the number of affected eyes with mild-to-moderate score peaked at 1 month and later declined without steroid treatment, although the manifestation was more persistent than aqueous cell infiltration (Figure 3B). Lens capsule deposits were noted after 0.5 months but quickly resolved (Figure 3D). There was no aqueous flare or vitreous haze detected over the 12.5-month study period, except grade 1+ vitreous haze observed in one eye at 12.5 months, corresponding to slight opacities without obscuration of retinal details.

Intraocular pressure (IOP) was assessed with tonometry at baseline and at 0.5, 1, 3, 6, 9, and 12.5 months post-dose. IOP remained normal in vehicle-treated eyes. There was a transient reduction in IOP in 4 of 14 eyes in the ADVM-022-treated group. This IOP reduction was observed in the eyes of animals that showed a higher grade of anterior chamber reaction at 0.5 months post-dosing (Figure 3A; animals A014 and A066). After reaching a nadir at 0.5 months, IOP improved toward normal at 1 month without anti-inflammatory treatment and returned to baseline levels by 3 months, where it remained for the duration of the study (Figure 3F).

The average retinal thickness and volume was assessed by spectral domain optical coherence tomography (SD-OCT) in the region defined by the grid for the early treatment of diabetic retinopathy study (ETRDS; ClinicalTrials.gov: NCT00000151), including the foveal region. Retinal thickness and retinal volume did not change over the 12.5 months following IVT delivery of ADVM-022 (Figure 4), indicating that there was no significant retinal edema during the observation period and that the continuous exposure of aflibercept to the animals did not induce gross degenerative retinal structural changes. In addition, fundoscopy (baseline; 0.5, 1, 3, 6, 9, and 12.5 months) and fluorescein angiography (baseline; 3, 6, 9, and 12.5 months) did not identify changes in retinal morphology, optic nerve head, or vascular integrity (Figure S3).

Long-Term Efficacy of ADVM-022 in the Laser-Induced CNV Model

Initially, the efficacy of ADVM-022 was evaluated in a short-term laser-induced CNV study when ADVM-022 was delivered by IVT injection 56 days prior to the laser challenge. This study found that ADVM-022 was as effective in suppressing laser-induced exudative lesions as aflibercept, administered immediately after laser treatment (Figure S4).

A subsequent long-term study addressed the durability of ADVM-022 efficacy in the CNV model. Four NHPs that received ADVM-022 and four that received vehicle 13 months prior were randomly chosen in their respective groups to undergo laser photocoagulation in the peri-macular region of the retina to induce CNV lesions (Table 1; groups 1a and 2a). A group of four additional naive animals received a single IVT injection of 1.2 mg commercially available aflibercept recombinant protein at the time of photocoagulation to serve as a positive control (group 3). The amount of aflibercept recombinant protein injected corresponded to clinical doses used in patients but adjusted proportionately to the vitreal volume of the African green monkey eye (half human vitreous volume). Efficacy was assessed as the frequency of clinically relevant grade IV exudative lesions (defined as bright hyperfluorescence early or midtransit, with late fluorescein leakage extending beyond the borders of the laser spot). Previous NHP studies have demonstrated that the incidence

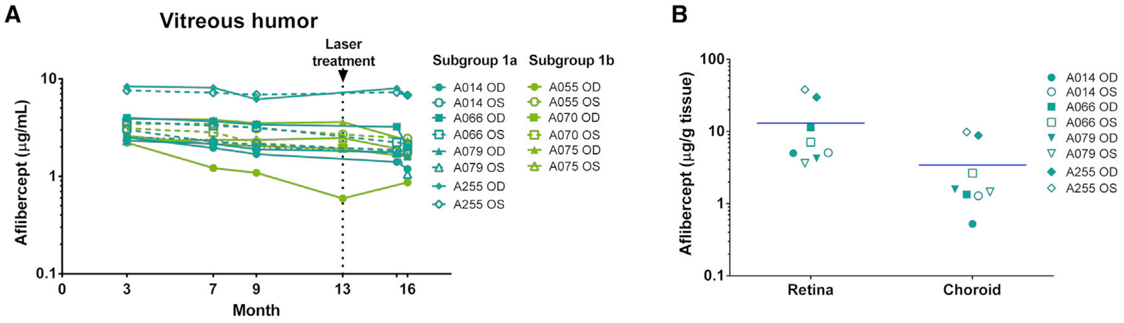

Figure 2. IVT Delivery of ADVM-022 (2×10^{12} vg/Eye) Provides Stable Intraocular Expression of Aflibercept

Seven adult monkeys (group 1, Table 1) were given an IVT injection of ADVM-022. Aflibercept expression levels were measured in vitreous humor samples A) collected according to the schedule in Table 2, as well as from both retina and choroid tissue collected 16 months after IVT injection (B). (A) Teal, lasered eyes; green, non-lasered eyes.

of grade IV lesions, as a component within the full angiogram grading scale, is robustly representative of the response to the IVT injection of standard-of-care anti-angiogenic drugs.[23,24] In this study, the grade IV lesions were assessed at 2 and 4 weeks post-laser by fluorescein angiography, and the conclusions were supported by cross-sectional measures of CNV complexes generated by OCT imaging.

Although nine laser spots were applied in each eye, not all lesions were assessable due to the presence of hemorrhage masking the lesion sites (a common occurrence following laser photocoagulation)[23] or poor visibility due to insufficient mydriasis (Table S1). A large subretinal hemorrhage formed in two eyes during lasering procedure made laser photocoagulation infeasible in a total of four neighboring lesions.

Consistent with this model, the incidence of grade IV lesions was 43% and 40% in the vehicle group at 2 and 4 weeks post-laser, respectively (Figure 5, right panel). The aflibercept recombinant protein control group had a statistically significant lower incidence of grade IV lesions than the vehicle-treated group at 2 and 4 weeks post-laser (3% and 5%, respectively $p < 0.0001$, Figure 5). In comparison, the animals that received ADVM-022 also had a significantly lower incidence of grade IV lesions than vehicle animals at 2 and 4 weeks post-laser (0% and 6%, respectively; $p < 0.0001$, Fisher's exact test). No statistical difference in the grade IV lesion incidence was observed between ADVM-022 and aflibercept recombinant protein groups.

To address the potential eye-to-eye variability in response to the treatments, all groups were also evaluated based on the counts of grade IV lesions per eye. This analysis confirmed a statistically significantly lower incidence of grade IV lesions in the eyes treated with ADVM-022 compared with vehicle ($p < 0.05$ at 2 weeks post-laser and 4 weeks post-laser, Mann-Whitney U test). Treatment with aflibercept recombinant protein at the time of lesion also resulted in a statistically significant decrease in the incidence of grade IV lesions compared with vehicle at 4 weeks post-laser ($p < 0.05$). There was no significant difference in the incidence of grade IV lesions between the ADVM-022 and aflibercept recombinant protein-treated eyes at 2 or 4 weeks post-laser ($p = 0.4$ and 0.85, respectively) (Figure S5).

To explore whether the suppression of the number of grade IV exudative lesions corresponded with the decrease in the size of CNV fibrovascular complexes, we used SD-OCT to assess the anatomic appearance of CNV lesions. SD-OCT has become a valuable complement to fluorescein angiography and associated CNV leakage grading scales, to characterize wAMD in clinical and preclinical evaluations to measure the response to the anti-angiogenic therapies. By providing cross-sectional images of the retina,[25–28] SD-OCT has been demonstrated to be a reliable metric for the evaluation of CNV complex size.[27,29] SD-OCT reveals CNV complex morphology that correlates with histological data and has been successfully applied in the NHP model of CNV to test the efficacy of novel anti-angiogenic agents.[30,31]

Subretinal fibro-vascular complexes in CNV were identified as hyperreflective zones in the SD-OCT section images, as described in the Materials and Methods. CNV complex formation was quantified in OCT images collected at 2 and 4 weeks post-laser photocoagulation, with each site evaluated by area analysis of cross-sectional OCT images. IVT ADVM-022 or aflibercept recombinant protein treatment resulted in a comparable and statistically significant reduction in the CNV complex area at 2 and 4 weeks compared with the vehicle group. While the mean CNV complex area measured from principal axis images was 142,369 and 82,923 μm^2 in the vehicle group at 2- and 4-weeks post-laser, respectively, the mean CNV complex area in ADVM-022 treated eyes was 45,078 and 23,792 μm^2 at 2 and 4 weeks, respectively. Similarly, the mean CNV complex area measured in the aflibercept-treated group was 44,503 and 26,622 μm^2 at 2 and 4 weeks, respectively ($p < 0.0001$ at both time points for ADVM-022 or aflibercept recombinant protein versus vehicle; Figure 6). There were no significant differences between ADVM-022 and aflibercept recombinant protein groups at 2 or 4 weeks post-laser ($p > 0.999$ at both time points).

DISCUSSION

Anti-angiogenic recombinant protein therapies targeting VEGFA such as aflibercept,[6] ranibizumab,[4,32] and bevacizumab[1,5] have shown to be very effective at reducing CNV and vision loss associated with wAMD. However, compliance with the treatment regimen for these therapies can constitute a considerable burden to patients[7] and a

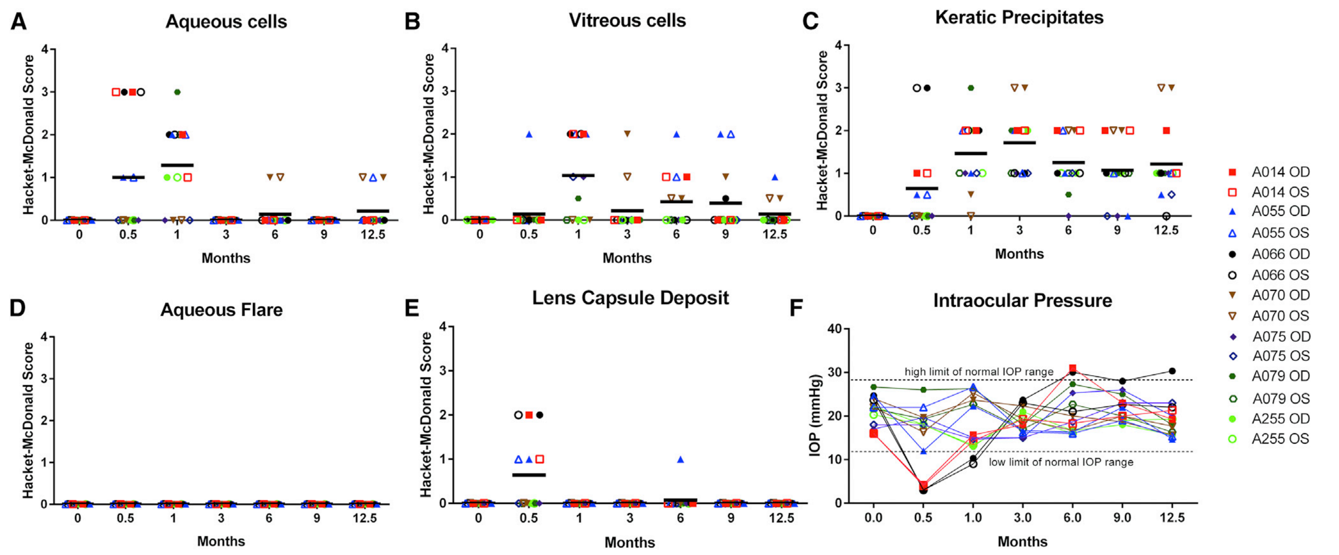

Figure 3. ADVM-022 Has Generally Mild and Transient Effects on Aqueous and Vitreous Cell Infiltrates, Keratic Precipitates, Lens Capsule Deposits, and Intraocular Pressure

The parameters scored by the Hackett-McDonald irritation and inflammation scoring system are shown: aqueous cells (A), vitreous cells (B), keratic precipitates (C), aqueous flare (D), lens capsule deposits (E), and IOP (F) in ADVM-022 IVT-injected eyes. No vitreous haze was detected except in one eye at 12.5 months (grade 1+). Horizontal bars show mean values (n = 14 eyes). The decrease in IOP coincides with a peak in markers of inflammation. Each symbol represents one individual eye.

survey of 90 retinal specialists with large clinical practices determined that reduced dosing frequency is the greatest unmet need in wAMD therapy.[33] Novel approaches to extend the benefits of anti-VEGFA therapy are being evaluated, including longer-acting anti-VEGFA agents, port delivery, and injectable depot systems for delivering anti-VEGFA therapies, topical eye-drops, and oral anti-VEGFA therapies.[34] While these approaches may reduce or eliminate the need for ocular injections, they provide finite amounts of drug for a chronic disease that requires long-term, if not life-long therapy. Gene-based therapy for wAMD has the potential to overcome current compliance issues by ideally providing a life-long supply of anti-VEGFA protein at the site of the disease following a single vector administration.

Route of administration is an important consideration when developing therapies administered via ocular injection. Subretinal administration of recombinant AAV (rAAV) vectors is a very efficient means to deliver therapeutic genes to the retina, and this route of administration is currently used in an approved gene therapy to treat a rare form of genetic blindness (Leber's congenital amaurosis type 2 [LCA2]).[35] Unlike wAMD, a significant clinical benefit in this indication can be achieved by transducing a relatively small number of cells. Given that subretinal injection is limiting with respect to the amount of vector that can be infused and the localized transduction of cells under the bleb, with little diffusion of the vector beyond the bleb boundaries,[36] this delivery method may be suboptimal for wAMD gene therapy. Nonetheless, one clinical trial was conducted to evaluate the efficacy of an AAV2 vector expressing sFlt-1 using this route of administration.[37] Another trial is also currently underway with anti-VEGFA Fab fragment expression cassette delivered via subretinal injection with an AAV8 vector that has shown better transduction efficiency than AAV2[38] (ClinicalTrials.gov: NCT03066258). Other potential drawbacks of subretinal injection include risks such as development of cataracts[39] and the requirement for a surgical setting (unlike IVT administration, which can be performed as an outpatient procedure in the doctor's office). Currently, only seven prequalified centers in the United States offer the gene therapy for LCA2, suggesting that access to other gene therapies that require subretinal injection may be limited.

Owing to its improved transduction efficiency of the retina when compared to AAV2,[18] IVT administered AAV2.7m8 has the potential to enable high levels of aflibercept expression. This is supported by the data described in Figure S1 demonstrating robust retinal transduction in NHPs following IVT administration of the AAV2.7m8 capsid carrying the GFP cDNA. At a dose of 5×10^{11} vg/eye the pattern of transduction with AAV2.7m8-GFP in African green monkey eye was identical to that described for this vector in cynomolgus monkey (Figure S1).[18] The vector administration resulted in robust transduction in the foveal pit, with low to no expression in peri-macular region extending to the vascular arcades (due to the presence of the thick inner limiting membrane in this area) and increased density of GFP-expressing cells toward the peripheral retina, with high levels of transduction in perivascular areas. Similarly, ADVM-022 is expected to robustly transduce a larger portion of the retina, allowing for an increased number of cells to produce aflibercept and contribute to the efficacy of the treatment.

Results presented here demonstrate that IVT injection of ADVM-022 provides stable and robust intraocular expression of aflibercept and results in high levels of unbound aflibercept within the tissue in which

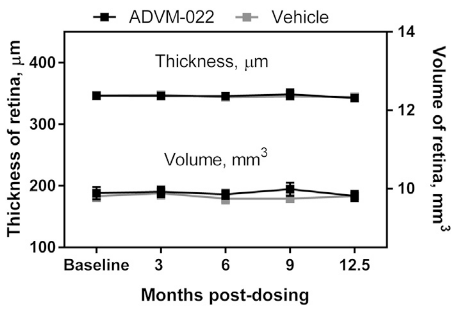

Figure 4. Prolonged Exposure of Retina to Aflibercept following ADVM-022 Administration Does Not Result in Changes in Retinal Volume or Thickness

The average retinal thickness and volume was assessed by SD-OCT in the region defined by the grid for the early treatment of diabetic retinopathy study (ETRDS) including the foveal region. Measurements were taken in the ADVM-022 and vehicle treated groups at baseline, 3, 6, 9, 12.5 months post-dosing. n = 14 eyes. Mean values ± SEM shown.

wAMD occurs, i.e., retina and choroid (Figure 2). Importantly, aflibercept levels in the aqueous humor of NHPs measured 7 months following IVT administration of ADVM-022 were similar to aflibercept levels in the aqueous humor of human subjects measured 1 month following bolus monthly or bi-monthly administration of aflibercept protein.[40] At this time point, human patients had a significantly reduced free VEGFA-A levels in the aqueous humor.[40] This suggests that AAV2.7m8-based gene therapy may provide sustained therapeutic levels of aflibercept expression to the eye. In the current study, a single IVT injection of ADVM-022 administered 13 months prior to laser photocoagulation reduced the incidence of grade IV lesions to the same extent as a 1.2 mg IVT bolus of aflibercept recombinant protein, administered at the time of lesioning (Figures 5 and S5), as measured by FA. The effect of the treatment on grade IV CNV lesion incidence correlated with smaller size of fibrovascular CNV complexes, as measured by SD-OCT. Multiple preclinical and clinical studies using AAV-based gene therapy approaches, particularly in the eye, have shown transgene product expression lasting several years.[35,41] Similarly, as judged by the robust aflibercept expression in retinal tissues obtained at 16 months post-injection, it is likely that cells transduced with ADVM-022 will continue to generate aflibercept for an extended period of time.

Intraocular inflammation following the IVT administration of treatments for ocular diseases has been reported in other preclinical studies of retinal gene therapy[42,43] and, if severe or prolonged, could limit the use of such therapies in the clinic. In our early preclinical studies, we had found a favorable ocular safety profile with AAV2.7m8 vectors administered by IVT injection at doses up to 2×10^{12} vg/eye,[44] which provided rationale for the use of this dose to evaluate ADVM-022 long- term efficacy (data not shown). The assessments of inflammatory response (aqueous cells, vitreous cells, aqueous flare, vitreous haze, and keratic precipitates) and IOP presented here (Figure 3) show that the mild to marked effects of ADVM-022 on aqueous cells, aqueous flare, and vitreous haze were generally transient and self-resolving, with a peak response at approximately 1-month post-dose with partial resolution by 3-months post-dose. However, vitreous cell infiltrates and keratic precipitates of diminished size and more pigmented composition, were present at 12.5 months post-dose. Longer persistence of keratic precipitates and vitreous cell compared to anterior chamber cell likely reflects a more dynamic turnover of the of inflammatory mediators, cells and debris in the aqueous chamber, while the corneal endothelial surface and vitreous chamber present a more static environment, resulting in slower clearance of remnant cellular and acellular material. As such, persisting keratic precipitates in this study appeared to be a reflection of the clearance rate of the cellular component of the initial inflammatory event.

The potential for ADVM-022 to induce ocular inflammation in patients can be mitigated by treating with anti-inflammatory agents, as has been successful in previous clinical trials of IVT-administered AAV vectors.[45] For instance, in a previous clinical trial of AAV2-sFlt-1, keratic precipitates that developed in a patient 1 month after receiving the AAV2-based gene therapy were successfully treated with topical steroids.[15] This suggests that this potential side effect would not be rate limiting with respect to clinical development of ADVM-022.

Long-term VEGFA suppression has been cited as a risk of degeneration of retinal tissue (geographic atrophy [GA]), as studies have shown a requirement for VEGFA in the physiological integrity of the retina.[46,47] Direct evidence linking anti-VEGFA therapy to GA, however, remains to be established. The results presented here show that the robust and sustained long-term expression of aflibercept more than 12 months post-IVT administration of ADVM-022 did not result in changes to macular volume or thickness as assessed by SD-OCT (Figure 4) or changes to retinal or optic nerve head morphology or vascular integrity (Figure S3). In addition, in a separate study a 12-month evaluation of retinal function by electroretinography (ERG) in human AMD patients treated with IVT injections of ranibizumab did not reveal any functional decline,[48] and retinal or retina pigmented epithelium atrophy and other serious ocular adverse events have not been observed in clinical trials of other gene-based approaches to VEGFA inhibition at 12 months.[14,15,37]

The results presented here demonstrate for the first time preclinical evidence of long-term robust expression and efficacy of aflibercept, an approved anti-VEGFA protein therapy for wAMD delivered by IVT administration of a gene-therapy vector. Aflibercept levels found in the vitreous and retinal tissue of NHPs reported here are consistent with therapeutic levels of the protein shortly after bolus injection of aflibercept protein in rabbits.[12] This sustained expression and IVT route of administration, which does not require specialized surgical skills and bypasses the risks associated with subretinal injection, are important factors that could address the current compliance challenges associated with protein therapies for wAMD. ADVM-022 combines the AAV2.7m8 capsid, which has improved retinal transduction following IVT administration, and a strong ubiquitous expression cassette, which offers important advantages over prior and ongoing gene therapy approaches. The robust pharmacology

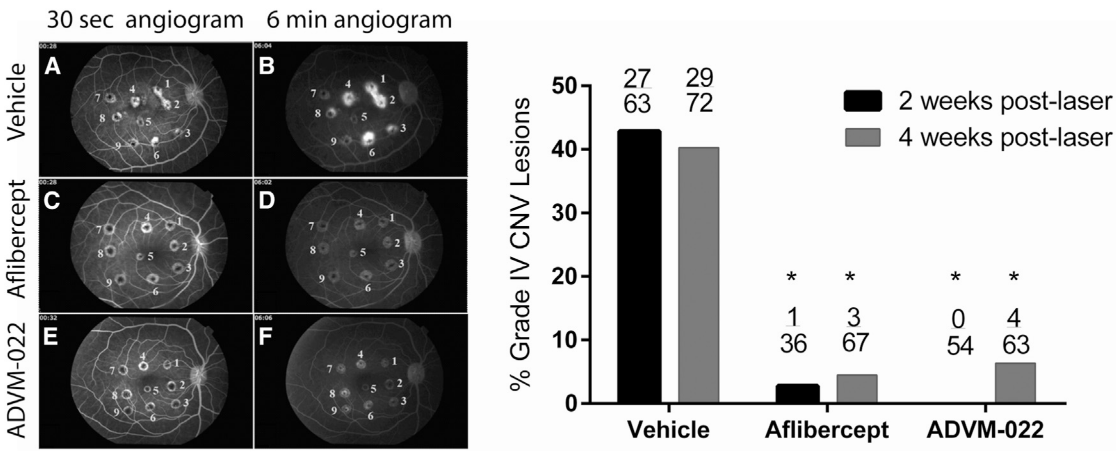

Figure 5. Single-Dose IVT ADVM-022 Significantly Reduces the Incidence of Grade IV Lesions When Administered 13 Months Prior to Laser-Induced CNV
Left, Representative early (~30 s; A, C, and E)- and late-phase (~6 min; B, D, and F) fluorescence angiograms from vehicle-injected (A and B), aflibercept-injected (C and D), and ADVM-022-injected eyes (E and F). Right, Incidence of grade IV lesions in groups treated 13 months prior to the CNV induction with vehicle or ADVM-022 or treated with aflibercept immediately after the laser photocoagulation. Numbers on the top of bars show the absolute number of grade IV lesions scored over the total number of assessable lesions. $p < 0.0001$ versus vehicle (Fisher's exact probability test). There was no statistical difference between the ADVM-022 and aflibercept groups.

and pharmacokinetics results reported here support the initiation of ADVM-022 clinical trials in patients with wAMD.

MATERIALS AND METHODS

Vector

ADVM-022 utilizes the AAV2.7m8 capsid, a variant of AAV2 that includes a 10-amino-acid insertion in loop IV of the AAV2 viral protein 3 (VP3).[18] The DNA genome consists of the viral inverted terminal repeats from AAV2 flanking the expression cassette, C11.CO.aflibercept. This C11.CO.aflibercept cassette consists of regulatory elements including the human cytomegalovirus (CMV) immediate-early enhancer and promoter, an adenovirus tripartite leader sequence (TPL) followed by an enhancer element from the major late promoter (eMLP), a synthetic intron, and a Kozak sequence driving expression of aflibercept. cDNA of aflibercept is followed by a human scaffold attachment region (SAR) and the human growth hormone (GH) polyadenylation site. Aflibercept is a recombinant chimeric protein consisting of the VEGFA binding portion of human VEGFR-1 (domain 2) and VEGFR-2 (domain 3 or KDR) fused to the Fc portion of human IgG1 immunoglobulin.

Aflibercept

Aflibercept recombinant protein used as positive control is manufactured by Regeneron Pharmaceuticals and was purchased commercially.

Animals and Study Design

This study was conducted in naive adult (5–12 years old) African green monkeys (*Chlorocebus sabaeus*) of both sexes (N = 18; randomized into three groups by weight; Table 1). All animals were used in accordance with the ARVO Statement for the Use of Animals in Ophthalmic and Vision Research. The use of bilateral treatments and all procedural aspects of the animal studies was approved by the Animal Care and Use Committee overseeing animal welfare at the primate facility (St. Kitts Biomedical Research Foundation, St. Kitts, West Indies) with which RxGen (Connecticut) maintains a facility use agreement. All animals were in the normal range at baseline ophthalmic screening, including tonometry, slit lamp biomicroscopy, fundoscopy, color fundus photography (CFP), fluorescein angiography (FA), and OCT. Groups 1 and 2 were enrolled on study on day 0, while group 3a was enrolled at 12.5 months.

Animal Care and Handling

Animals were anesthetized for all procedures and ophthalmic evaluations (8.0 mg/kg ketamine/1.6 mg/kg xylazine, intramuscularly [IM] to effect). General well-being was assessed before, during, and after sedation as well as twice daily on non-procedure days. The amount of food biscuits consumed daily was monitored. Body weight was measured at the time of ophthalmic examinations.

Test Article Administration

On study day 0 groups 1 and 2 received bilateral (OU) IVT injections of 50-μL ADVM-022 or vehicle (Table 1). Group 3 animals received IVT 1.2 mg (30 μL) aflibercept (Eylea, 40 mg/mL, approved therapy for wAMD) OU following laser treatment at 13 months. IVT doses were administered under local anesthesia (0.5% proparacaine) using a 31G 5/16-inch needle (Ulticare Vet RX U-100, # 09436) 2 mm posterior to the limbus in the inferior temporal quadrant, targeting the central vitreous.

Laser-Induced CNV

At 13 months post-dose, CNV was induced between the temporal vascular arcades by laser photocoagulation to the "a" subgroups (Table 1). Nine laser spots were symmetrically placed in each eye with an Iridex Oculight TX 532 nm laser. CFP was performed immediately after the laser treatment to document the laser lesions.

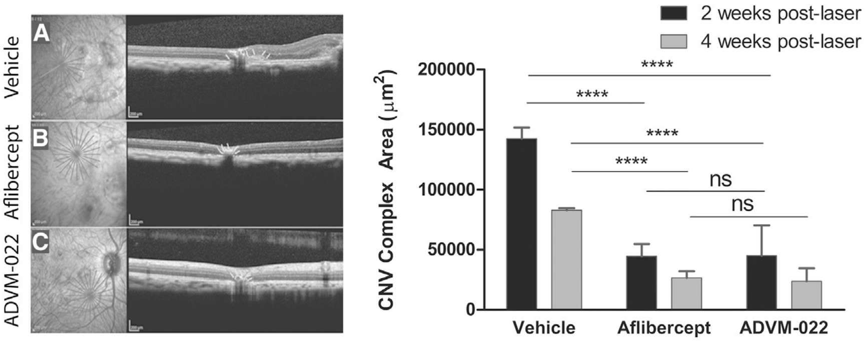

Figure 6. Single Dose of IVT ADVM-022 Administered 13 Months Prior to Laser Significantly Reduces the Size of Fibro-vascular CNV Complexes
Left, Representative OCT images at 4 weeks post-laser from eyes receiving vehicle (A), aflibercept (B), and ADVM-022 (C). Right, Size of CNV complex evaluated at 14 and 28 days post-laser photocoagulation. CNV complex size is presented as the area of the CNV in the optical cross-section. Mean CNV complex areas were significantly smaller in the ADVM-022 and aflibercept groups compared with vehicle. Data shows mean maximum cross-sectional CNV area with bars indicating SEM (7 to 8 eyes analyzed per group). Number of lesions assessed (n) was 71, 68, and 62 for vehicle-, aflibercept-, and ADVM-022-treated groups, respectively, at 2 weeks post-laser and 70, 67, and 63 4 weeks post-laser. There was no statistical difference between the ADVM-022 and aflibercept groups. (****$p < 0.0001$; ns, non-significant; Mann-Whitney U test).

Animals with areas demonstrating severe retinal or subretinal hemorrhage immediately post-laser that did not resolve by the time of follow-up examination were excluded from quantitative image analyses.

Follow-Up Evaluations

All group 1 and 2 eyes were examined by slit lamp biomicroscopy, fundoscopy, tonometry, and CFP at baseline, 14 days, and 1, 3, 6, 9, and 12.5 months post IVT injection. Slit lamp biomicroscopy and fundoscopy were additionally conducted immediately prior to laser at 13 months. FA and OCT were conducted at baseline and 3, 6, 9, and 12.5 months post-dose. All eyes were examined by slit lamp biomicroscopy, fundoscopy, tonometry, CFP, FA, and OCT at 2 and 4 weeks post-laser-photocoagulation.

Evaluations

Slit Lamp Biomicroscopy and Fundoscopy

Anterior segment cells and flare were examined by slit lamp biomicroscopy (SL-2E, Topocon) and scored using a modified Hackett-McDonald scale. Evaluation of the posterior wall and vitreous inflammation was performed by posterior segment slit lamp exam employing a 90-diopter lens. Retinal infiltrates and hemorrhage, vascular dilation, tortuosity, and sheathing, and optic disc edema were also evaluated during the fundoscopy.

CFP and FA

Bilateral color fundus images were captured using a Topcon TRC-S0EX retinal camera with Canon 6D digital imaging hardware and New Vision Fundus Image Analysis System software under pupil dilation (10% phenylephrine hydrochloride and 1% cyclopentolate hydrochloride). FA was performed with 10% sodium fluorescein (0.1 mL/kg, IV). FA in oculus dexter (OD) preceded angiography in oculus sinister (OS) by 4–6 hours to allow washout of the fluorescein between the angiogram image series (0, 2, 15, 20, 25, 30, 40, and 50 s, 1, 2, 3 and 6 min). Graded scoring of angiograms was performed on fluorescein angiogram series collected at 2 and 4 weeks post-laser by a masked investigator.

Fluorescein leakage was graded by a masked investigator using the following grading scale: I, no hyperfluorescence; II, hyperfluorescence without leakage and no significant residual staining in late-phase angiograms; III, hyperfluorescence early or mid-transit with late leakage and significant residual staining; IV, hyperfluorescence early or mid-transit with late leakage extending beyond the borders of the treated area.

OCT

OCT was performed every 3 months to evaluate retinal thickness, volume, and structural integrity using a Heidelberg Engineering Spectralis OCT Plus system (Vista, CA) and Heidelberg Eye Explorer (HEYEX) software (v1.6.1.). The dense retinal volume scans consisted of 48 parallel scans of 30° in the horizontal plane positioned 50 μm apart with image averaging over 50 automatic retinal tracking (ART) frames with scan grid centered on the fovea. Retinal thickness and volume were calculated by the OCT system at time points prior to laser treatment. To measure laser-induced CNV size using cross-sectional area analysis of the lesion, nine star-shaped scans per eye, centered on each lesion, were performed. Star-shaped scans consisted of 12 scans intersecting at 15° in a clock pattern with image averaging over 25 ART frames. The principal axis of maximal CNV complex formation and corresponding 60° and 120° axes were defined within each star-shaped scan at each laser lesion by the OCT examiner and exported for analysis. The CNV complex area was measured by a masked evaluator using ImageJ to delineate the CNV complex boundary and calculate maximum complex area in square microns (μm^2) and maximum thickness in microns within each exported OCT section.

Tonometry

IOP measurements were made with a TonoVet (iCare, Finland) tonometer at the dog (d) setting. Under full sedation, monkeys were placed in a supine position in an IOP testing apparatus. Three measures were taken per eye at each time point and the mean value calculated.

Vitreous Humor Collection

Vitreous humor (50 μL each in-life/50 μL × 3 at termination) was sampled at baseline, 3, 7, 9 months (subgroups "a" and "b"), 15.5 months (prior to sacrifice, "a" subgroups), and 13 months

("b" subgroups). Samples were collected using a 0.3 mL insulin syringe with a 27G needle introduced aseptically 3 mm posterior to the limbus.

Termination and Tissue Collection

All "a" subgroup animals were euthanized with pentobarbital after completion of final ophthalmic examinations and confirming the quality of fundus imaging at 15.5 months. Prior to sacrifice, samples of vitreous humor, serum, and plasma were collected. Following sample collection, animals were euthanized with pentobarbital and globes enucleated. Globes were dissected to isolate cornea, iris-ciliary body, lens, vitreous (full volume remaining after initial tap), choroid, retina, sclera, and optic nerve.

Bioanalysis for Aflibercept Expression

Unbound aflibercept levels in vitreous humor, retina, and choroid were measured using a quantitative ELISA. ELISA plates (NUNC MaxiSorp) were coated with 100 μL/well of recombinant human VEGFA (rhVEGFA) (R&D Systems) at a concentration of 1 μg/mL in coating buffer (R&D Systems) and incubated overnight at 4°C. After washing with wash buffer (KPL), the plates were blocked with 300 μL/well of protein-free blocking buffer (Pierce). Afterward, the plates were washed and the samples were added (100 μL/well) at following dilutions (1:1,000 for ADVM-022-treated samples and 1:50 for vehicle-treated samples) and incubated for 2 hr at room temperature (RT). The plates were then washed again, and 100 μL/well of anti-human Fc domain of IgG (Fcγ)-specific antibody conjugated to horseradish peroxidase (HRP) (Jackson ImmunoResearch) at 500 ng/mL in BSA 1% in PBS was added to the wells. After washing, 100 μL/well of SuperSignal ELISA Pico Chemiluminescent Substrate (Thermo Fisher Scientific) was added to the wells, and luminescence signal was measured using a microplate luminometer.

Data Analysis

Graded (I–IV) scoring of laser lesions was analyzed using the Fisher's exact test where incidence of grade IV lesions was assigned "Yes" and any other grading assigned "No." To address the potential eye-to-eye variability, all groups were also evaluated based on the counts of grade IV lesions per eye using Mann-Whitney U test. CNV complex cross-section area data were not of normal distribution (D'Agostino-Pearson omnibus normality test) and were analyzed by Mann-Whitney test as well. All statistics were performed using the statistical analysis software GraphPad Prizm. Values of $p < 0.05$ or smaller were considered statistically significant.

SUPPLEMENTAL INFORMATION

Supplemental Information includes Supplemental Materials and Methods, one table, and five figures and can be found with this article online at https://doi.org/10.1016/j.ymthe.2018.11.003.

AUTHOR CONTRIBUTIONS

Conceptualization, M.B., S.K., M.G..; Investigation, R.G., P.S., A.K., J.G.; Visualization, B.V.; Data Curation, R.G., B.V.; Data Analysis, B.V.; Formal Analysis, R.G.; Writing – Review & Editing, R.G., B.V., P.S., A.K., J.G., C.G., M.B., S.K., M.G., M.L., W.H.; Supervision, C.G., M.G.; Project Administration, C.G., R.G., B.V. contributed equally.

CONFLICTS OF INTEREST

C.G., M.G., A.K., J.G., P.S., B.V., and R.G. are employees of Adverum Biotechnologies and hold stock grants; M.B. holds a grant of Adverum Biotechnologies stock; S.K. is a paid consultant of Adverum Biotechnologies and holds a grant of Adverum Biotechnologies stock.

ACKNOWLEDGMENTS

This study was funded by Adverum Biotechnologies, Inc. The authors thank S. Seiler for editorial assistance; J. Nieves, A. Nguyen, and R. Rosario for technical support; and A. Ramirez and M. Steel for test article production and quality control. We also thank A. Phillips for insights and helpful suggestions during the development of this project.

REFERENCES

1. Avery, R.L., Pearlman, J., Pieramici, D.J., Rabena, M.D., Castellarin, A.A., Nasir, M.A., Giust, M.J., Wendel, R., and Patel, A. (2006). Intravitreal bevacizumab (Avastin) in the treatment of proliferative diabetic retinopathy. Ophthalmology *113*, 1695.e1–1695.e15.
2. Hernández-Zimbrón, L.F., Zamora-Alvarado, R., Ochoa-De la Paz, L., Velez-Montoya, R., Zenteno, E., Gulias-Cañizo, R., Quiroz-Mercado, H., and Gonzalez-Salinas, R. (2018). Age-Related Macular Degeneration: New Paradigms for Treatment and Management of AMD. Oxid. Med. Cell. Longev. *2018*, 8374647.
3. Ferrara, N. (2010). Vascular endothelial growth factor and age-related macular degeneration: from basic science to therapy. Nat. Med. *16*, 1107–1111.
4. Rosenfeld, P.J., Rich, R.M., and Lalwani, G.A. (2006). Ranibizumab: Phase III clinical trial results. Ophthalmol. Clin. North Am. *19*, 361–372.
5. Lynch, S.S., and Cheng, C.M. (2007). Bevacizumab for neovascular ocular diseases. Ann. Pharmacother. *41*, 614–625.
6. Heier, J.S., Brown, D.M., Chong, V., Korobelnik, J.F., Kaiser, P.K., Nguyen, Q.D., Kirchhof, B., Ho, A., Ogura, Y., Yancopoulos, G.D., et al.; VIEW 1 and VIEW 2 Study Groups (2012). Intravitreal aflibercept (VEGF trap-eye) in wet age-related macular degeneration. Ophthalmology *119*, 2537–2548.
7. Boyle, J., Vukicevic, M., Koklanis, K., Itsiopoulos, C., and Rees, G. (2018). Experiences of patients undergoing repeated intravitreal anti-vascular endothelial growth factor injections for neovascular age-related macular degeneration. Psychol. Health Med. *23*, 127–140.
8. Boulanger-Scemama, E., Querques, G., About, F., Puche, N., Srour, M., Mane, V., Massamba, N., Canoui-Poitrine, F., and Souied, E.H. (2015). Ranibizumab for exudative age-related macular degeneration: A five year study of adherence to follow-up in a real-life setting. J. Fr. Ophtalmol. *38*, 620–627.
9. Droege, K.M., Muether, P.S., Hermann, M.M., Caramoy, A., Viebahn, U., Kirchhof, B., and Fauser, S. (2013). Adherence to ranibizumab treatment for neovascular age-related macular degeneration in real life. Graefes Arch. Clin. Exp. Ophthalmol. *251*, 1281–1284.
10. Hussain, R.M., Hariprasad, S.M., and Ciulla, T.A. (2017). Treatment Burden in Neovascular AMD:Visual Acuity Outcomes are Associated With Anti-VEGF Injection Frequency. Ophthalmic Surg. Lasers Imaging Retina *48*, 780–784.
11. Holekamp, N.M., Liu, Y., Yeh, W.S., Chia, Y., Kiss, S., Almony, A., and Kowalski, J.W. (2014). Clinical utilization of anti-VEGF agents and disease monitoring in neovascular age-related macular degeneration. Am. J. Ophthalmol. *157*, 825–833.e1.
12. Park, S.J., Choi, Y., Na, Y.M., Hong, H.K., Park, J.Y., Park, K.H., Chung, J.Y., and Woo, S.J. (2016). Intraocular Pharmacokinetics of Intravitreal Aflibercept (Eylea) in a Rabbit Model. Invest. Ophthalmol. Vis. Sci. *57*, 2612–2617.

13. Bainbridge, J.W., Mistry, A., De Alwis, M., Paleolog, E., Baker, A., Thrasher, A.J., and Ali, R.R. (2002). Inhibition of retinal neovascularisation by gene transfer of soluble VEGF receptor sFlt-1. Gene Ther. *9*, 320–326.

14. Constable, I.J., Pierce, C.M., Lai, C.M., Magno, A.L., Degli-Esposti, M.A., French, M.A., McAllister, I.L., Butler, S., Barone, S.B., Schwartz, S.D., et al. (2016). Phase 2a Randomized Clinical Trial: Safety and Post Hoc Analysis of Subretinal rAAV.sFLT-1 for Wet Age-related Macular Degeneration. EBioMedicine *14*, 168–175.

15. Heier, J.S., Kherani, S., Desai, S., Dugel, P., Kaushal, S., Cheng, S.H., Delacono, C., Purvis, A., Richards, S., Le-Halpere, A., et al. (2017). Intravitreous injection of AAV2-sFLT01 in patients with advanced neovascular age-related macular degeneration: a phase 1, open-label trial. Lancet *390*, 50–61.

16. Lukason, M., DuFresne, E., Rubin, H., Pechan, P., Li, Q., Kim, I., Kiss, S., Flaxel, C., Collins, M., Miller, J., et al. (2011). Inhibition of choroidal neovascularization in a nonhuman primate model by intravitreal administration of an AAV2 vector expressing a novel anti-VEGF molecule. Mol. Ther. *19*, 260–265.

17. Rota, R., Riccioni, T., Zaccarini, M., Lamartina, S., Gallo, A.D., Fusco, A., Kovesdi, I., Balestrazzi, E., Abeni, D.C., Ali, R.R., and Capogrossi, M.C. (2004). Marked inhibition of retinal neovascularization in rats following soluble-flt-1 gene transfer. J. Gene Med. *6*, 992–1002.

18. Dalkara, D., Byrne, L.C., Klimczak, R.R., Visel, M., Yin, L., Merigan, W.H., Flannery, J.G., and Schaffer, D.V. (2013). In vivo-directed evolution of a new adeno-associated virus for therapeutic outer retinal gene delivery from the vitreous. Sci. Transl. Med. *5*, 189ra76.

19. Ashraf, M., and Souka, A.A.R. (2017). Aflibercept in age-related macular degeneration: evaluating its role as a primary therapeutic option. Eye (Lond.) *31*, 1523–1536.

20. Balaratnasingam, C., Dhrami-Gavazi, E., McCann, J.T., Ghadiali, Q., and Freund, K.B. (2015). Aflibercept: a review of its use in the treatment of choroidal neovascularization due to age-related macular degeneration. Clin. Ophthalmol. *9*, 2355–2371.

21. Hickey, D.G., Edwards, T.L., Barnard, A.R., Singh, M.S., de Silva, S.R., McClements, M.E., Flannery, J.G., Hankins, M.W., and MacLaren, R.E. (2017). Tropism of engineered and evolved recombinant AAV serotypes in the rd1 mouse and ex vivo primate retina. Gene Ther. *24*, 787–800.

22. Niwa, Y., Kakinoki, M., Sawada, T., Wang, X., and Ohji, M. (2015). Ranibizumab and Aflibercept: Intraocular Pharmacokinetics and Their Effects on Aqueous VEGF Level in Vitrectomized and Nonvitrectomized Macaque Eyes. Invest. Ophthalmol. Vis. Sci. *56*, 6501–6505.

23. Goody, R.J., Hu, W., Shafiee, A., Struharik, M., Bartels, S., López, F.J., and Lawrence, M.S. (2011). Optimization of laser-induced choroidal neovascularization in African green monkeys. Exp. Eye Res. *92*, 464–472.

24. Nork, T.M., Dubielzig, R.R., Christian, B.J., Miller, P.E., Miller, J.M., Cao, J., Zimmer, E.P., and Wiegand, S.J. (2011). Prevention of experimental choroidal neovascularization and resolution of active lesions by VEGF trap in nonhuman primates. Arch. Ophthalmol. *129*, 1042–1052.

25. Abri Aghdam, K., Pielen, A., Framme, C., and Junker, B. (2015). Correlation Between Hyperreflective Foci and Clinical Outcomes in Neovascular Age-Related Macular Degeneration After Switching to Aflibercept. Invest. Ophthalmol. Vis. Sci. *56*, 6448–6455.

26. Casalino, G., Bandello, F., and Chakravarthy, U. (2016). Changes in Neovascular Lesion Hyperreflectivity After Anti-VEGF Treatment in Age-Related Macular Degeneration: An Integrated Multimodal Imaging Analysis. Invest. Ophthalmol. Vis. Sci. *57*, OCT288–OCT298.

27. Giani, A., Luiselli, C., Esmaili, D.D., Salvetti, P., Cigada, M., Miller, J.W., and Staurenghi, G. (2011). Spectral-domain optical coherence tomography as an indicator of fluorescein angiography leakage from choroidal neovascularization. Invest. Ophthalmol. Vis. Sci. *52*, 5579–5586.

28. Keane, P.A., Liakopoulos, S., Jivrajka, R.V., Chang, K.T., Alasil, T., Walsh, A.C., and Sadda, S.R. (2009). Evaluation of optical coherence tomography retinal thickness parameters for use in clinical trials for neovascular age-related macular degeneration. Invest. Ophthalmol. Vis. Sci. *50*, 3378–3385.

29. Giani, A., Thanos, A., Roh, M.I., Connolly, E., Trichonas, G., Kim, I., Gragoudas, E., Vavvas, D., and Miller, J.W. (2011). In vivo evaluation of laser-induced choroidal neovascularization using spectral-domain optical coherence tomography. Invest. Ophthalmol. Vis. Sci. *52*, 3880–3887.

30. Cloutier, F., Lawrence, M., Goody, R., Lamoureux, S., Al-Mahmood, S., Colin, S., Ferry, A., Conduzorgues, J.P., Hadri, A., Cursiefen, C., et al. (2012). Antiangiogenic activity of aganirsen in nonhuman primate and rodent models of retinal neovascular disease after topical administration. Invest. Ophthalmol. Vis. Sci. *53*, 1195–1203.

31. Sidman, R.L., Li, J., Lawrence, M., Hu, W., Musso, G.F., Giordano, R.J., Cardó-Vila, M., Pasqualini, R., and Arap, W. (2015). The peptidomimetic Vasotide targets two retinal VEGF receptors and reduces pathological angiogenesis in murine and nonhuman primate models of retinal disease. Sci. Transl. Med. *7*, 309ra165.

32. Heier, J.S., Antoszyk, A.N., Pavan, P.R., Leff, S.R., Rosenfeld, P.J., Ciulla, T.A., Dreyer, R.F., Gentile, R.C., Sy, J.P., Hantsbarger, G., and Shams, N. (2006). Ranibizumab for treatment of neovascular age-related macular degeneration: a phase I/II multicenter, controlled, multidose study. Ophthalmology *113*, 633.e1–633.e4.

33. Cook, GP (2011). Reformulating Anti-VEGF for Extended-release Delivery in the Treatment of Retinal Diseases. Retina Today, March 2011, 70–72. http://retinatoday.com/2011/03/reformulating-anti-vegf-for-extended-release-delivery-in-the-treatment-of-retinal-diseases/.

34. Hussain, R.M., and Ciulla, T.A. (2018). Addressing the Anti-VEGF Treatment Burden. Rev. Ophthalmol. *25*, 20–24.

35. Russell, S., Bennett, J., Wellman, J.A., Chung, D.C., Yu, Z.F., Tillman, A., Wittes, J., Pappas, J., Elci, O., McCague, S., et al. (2017). Efficacy and safety of voretigene neparvovec (AAV2-hRPE65v2) in patients with RPE65-mediated inherited retinal dystrophy: a randomised, controlled, open-label, phase 3 trial. Lancet *390*, 849–860.

36. Bruewer, A.R., Mowat, F.M., Bartoe, J.T., Boye, S.L., Hauswirth, W.W., and Petersen-Jones, S.M. (2013). Evaluation of lateral spread of transgene expression following subretinal AAV-mediated gene delivery in dogs. PLoS ONE *8*, e60218.

37. Rakoczy, E.P., Lai, C.M., Magno, A.L., Wikstrom, M.E., French, M.A., Pierce, C.M., Schwartz, S.D., Blumenkranz, M.S., Chalberg, T.W., Degli-Esposti, M.A., and Constable, I.J. (2015). Gene therapy with recombinant adeno-associated vectors for neovascular age-related macular degeneration: 1 year follow-up of a phase 1 randomised clinical trial. Lancet *386*, 2395–2403.

38. Gao, G.P., Alvira, M.R., Wang, L., Calcedo, R., Johnston, J., and Wilson, J.M. (2002). Novel adeno-associated viruses from rhesus monkeys as vectors for human gene therapy. Proc. Natl. Acad. Sci. USA *99*, 11854–11859.

39. Ochakovski, G.A., Bartz-Schmidt, K.U., and Fischer, M.D. (2017). Retinal Gene Therapy: Surgical Vector Delivery in the Translation to Clinical Trials. Front. Neurosci. *11*, 174.

40. Celik, N., Scheuerle, A., Auffarth, G.U., Kopitz, J., and Dithmar, S. (2015). Intraocular Pharmacokinetics of Aflibercept and Vascular Endothelial Growth Factor-A. Invest. Ophthalmol. Vis. Sci. *56*, 5574–5578.

41. Constable, I.J., Lai, C.M., Magno, A.L., French, M.A., Barone, S.B., Schwartz, S.D., Blumenkranz, M.S., Degli-Esposti, M.A., and Rakoczy, E.P. (2017). Gene Therapy in Neovascular Age-related Macular Degeneration: Three-Year Follow-up of a Phase 1 Randomized Dose Escalation Trial. Am. J. Ophthalmol. *177*, 150–158.

42. Maclachlan, T.K., Lukason, M., Collins, M., Munger, R., Isenberger, E., Rogers, C., Malatos, S., Dufresne, E., Morris, J., Calcedo, R., et al. (2011). Preclinical safety evaluation of AAV2-sFLT01- a gene therapy for age-related macular degeneration. Mol. Ther. *19*, 326–334.

43. Ye, G.J., Budzynski, E., Sonnentag, P., Miller, P.E., Sharma, A.K., Ver Hoeve, J.N., Howard, K., Knop, D.R., Neuringer, M., McGill, T., et al. (2015). Safety and Biodistribution Evaluation in Cynomolgus Macaques of rAAV2tYF-CB-hRS1, a Recombinant Adeno-Associated Virus Vector Expressing Retinoschisin. Hum. Gene Ther. Clin. Dev. *26*, 165–176.

44. Woodburn, K.W., Ramirez, A., Steel, M., Vijay, S., Keravala, A., Barone, S.B., et al. (2016). Impact of vector design and administration technique in gene

therapy for the treatment of age- related macular degeneration. Hum. Gene Ther. *27*, A40.

45. Xue, K., Groppe, M., Salvetti, A.P., and MacLaren, R.E. (2017). Technique of retinal gene therapy: delivery of viral vector into the subretinal space. Eye (Lond.) *31*, 1308–1316.
46. Kurihara, T., Westenskow, P.D., Bravo, S., Aguilar, E., and Friedlander, M. (2012). Targeted deletion of Vegfa in adult mice induces vision loss. J. Clin. Invest. *122*, 4213–4217.
47. Saint-Geniez, M., Maharaj, A.S., Walshe, T.E., Tucker, B.A., Sekiyama, E., Kurihara, T., Darland, D.C., Young, M.J., and D'Amore, P.A. (2008). Endogenous VEGF is required for visual function: evidence for a survival role on müller cells and photoreceptors. PLoS ONE *3*, e3554.
48. Nishimura, T., Machida, S., Harada, T., and Kurosaka, D. (2012). Retinal ganglion cell function after repeated intravitreal injections of ranibizumab in patients with age-related macular degeneration. Clin. Ophthalmol. *6*, 1073–1082.

YMTHE, Volume *27*

Supplemental Information

Preclinical Evaluation of ADVM-022, a Novel Gene Therapy Approach to Treating Wet Age-Related Macular Degeneration

Ruslan Grishanin, Brian Vuillemenot, Pallavi Sharma, Annahita Keravala, Judith Greengard, Claire Gelfman, Mark Blumenkrantz, Matthew Lawrence, Wenzheng Hu, Szilárd Kiss, and Mehdi Gasmi

SUPPLEMENTAL INFORMATION

Supplemental Materials and Methods

AAV2.7m8-CMV-GFP Vector Description

AAV2.7m8-CMV-GFP utilizes the AAV2.7m8 capsid, a variant of AAV2 that includes a 10-amino acid insertion in Loop IV of the AAV2 viral capsid proteins (VP1-3). The DNA genome consists of the viral inverted terminal repeats (ITRs) from AAV2 flanking the expression cassette, CMV-GFP. This cassette includes the human cytomegalovirus (CMV) immediate early enhancer and promoter driving expression of enhanced green fluorescent protein (eGFP) followed by an SV40 poly adenylation (poly A) signal. The cassette also contains a synthetic intron 5` to the eGFP cDNA.

Expression following transuction of pig retinal explants with AAV.7m8-CMV-sFLT1 or AAV.7m8-C11-CO.sFLT

Pig neuroretina explants (n=3), placed flat in transwell inserts with the photoreceptor layer facing down, were transduced with either AAV.7m8-CMV-sFLT1 or AAV.7m8-C11-CO.sFLT at a multiplicity of infection (moi) of $2x10^4$. Explants were cultured in Neurobasal A media supplemented with B27 and L-Glutamine for 2 weeks with media changes every 2-3 days. Expression of secreted sFlt1 protein was measured in the 3 day-old culture medium collected at day 14 post-transduction. Concentration of secreted sFLT1 protein was analyzed using the Human VEGF R1/Flt-1 DuoSet ELISA from R&D systems.

cSLO imaging and immunofluorescence following AAV2.7m8-CMG-GFP vector transduction

The transduction efficiency in the African green monkey retina was evaluated by immunofluorescence 12 weeks post IVT injection of AAV2.7m8-CMV-GFP ($5x10^{11}$ vg/eye). The eyes were enucleated immediately after euthanasia and fixed by immersion in 4% paraformaldehyde for 24 hours at 4 ºC, and then transferred to 30% sucrose in phosphate buffer saline (PBS) followed by storage at 4 ºC. The retina was separated from the sclera and flatmounts were prepared by making 4 radial cuts at the periphery and covered with a coverslip using 30% sucrose solution in PBS. GFP fluorescence from the retinal flatmounts was imaged at 5X magnification upon excitation at 488nm; tiled images were collected and stitched together using Zeiss software (Carl Zeiss ZEN 2 Blue Edition).

Immunohistochemistry was performed on histological sections containing retina and RPE prepared by embedding tissue in frozen OCT, and sectioned at 7µm thickness prior to mounting on charged slides (VWR). Prior to incubation with the appropriate antibody, tissue sections were blocked for 1 hour at room temperature with 5% normal donkey serum (Jackson ImmunoResearch) and 6% bovine serum albumin (Amresco) in PBS (Gibco).

GFP was detected from the AAV2.7m8-CMV-GFP-trasduced cells using chicken polyclonal GFP antibody (Abcam) at 10µg/mL, followed by incubation with donkey anti-chicken IgY Alexa Flour 488 conjugated secondary antibody (Jackson ImmunoResearch) at 5µg/mL. To identify cone photoreceptors, rhodamine-conjugated peanut agglutinin (Vector Labs) was used at 5µg/mL. Retinal ganglion cells were stained with monoclonal anti-beta III tubulin (TUJ1) antibody (Abcam), at 5 µg/ml, and detected using donkey anti-mouse antibody conjugated with Alexa 647 (Thermo Scientific) at 5 µg/ml. Rod photoreceptors were stained using anti-rhodopsin 4D2 monoclonal antibody (Millipore) at 0.4µg/mL and detected with secondary donkey-anti-mouse antibody conjugated with Alexa Fluor 555 (ThermoFisher) at 5µg/mL. Nuclei were stained with DAPI Nucleic Acid Stain (Invitrogen), used at 0.5µM. Stained retinal sections were covered with a coverslip with mounting medium applied.

Supplemental Table 1: Summary of eyes/lesions excluded from scoring

Treatment	Animal	Number of lesions applied		Excluded eyes/ lesions	
		OD	OS	OD	OS
ADVM-022	A014	9	9		
	A255	9	9		
	A079	8	9	Eye excluded at post-laser week 2 and 4	Eye excluded at week 2 post-laser
	A066	9	9		
Vehicle	A260	9	9	Eye excluded at post-laser week 2	
	K973	9	9		
	A090	9	9		
	A191	9	9		
Aflibercept	A386	9	9		
	A540	9	9		Eye excluded at week 2 post-laser
	A678	9	9		Eye excluded from the scoring at week 2 post-laser
	A681	9	6	Eye excluded from the scoring at week 2 post-laser	Eye excluded from the scoring at week 2 post-laser; 2 lesions excluded at week 4 post-laser

Supplemental Figures

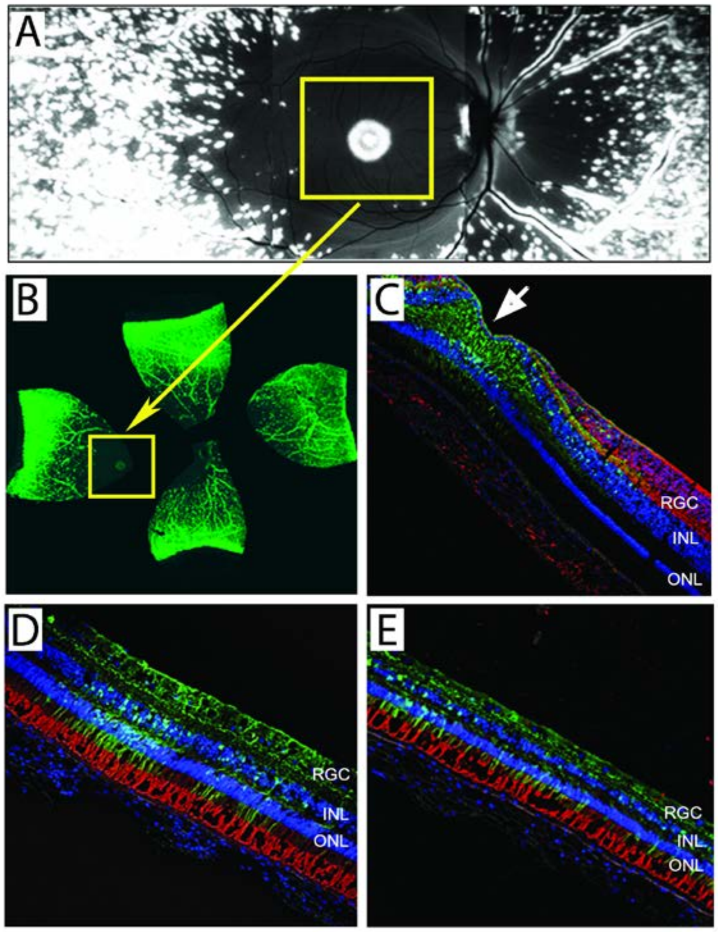

Figure S1. AAV2.7m8 driven GFP expression in the retina of African green monkey. AAV2.7m8-CMV-GFP ($5x10^{11}$ vg/eye) was injected IVT, and retinas were analyzed for the distribution of GFP expression 12 weeks post-injection.

A. Expression of GFP in the fundus of African green monkey imaged by confocal scanning laser ophthalmoscopy (cSLO). The box indicates GFP expression in the fovea. **B.** Retinal flat mount demonstrating GFP transgene expression in fovea, at the mid-periphery out to ora serrata. **C**. Retinal section through fovea and perifoveal area. White arrow indicates foveal pit. GFP is seen in cones, outer plexiform layer, sparse cells in inner nuclear layer (INL) and sparse retinal ganglion cells (RGC). (Red: TUJ-1, retinal ganglion cells). **D**. In mid-periphery, GFP expression is seen within the INL, inner plexiform layer of the retina, RGC layer and in patches of rod photoreceptors. (Red: rhodopsin, rod photoreceptors) **E.** In the periphery, GFP expression can be seen within the photoreceptor layer and the outer nuclear layer (ONL). (Red: rhodopsin). In all images green identifies GFP transgene expressing cells, and DAPI staining was used to identify cell nuclei. The disruptions in the outer segment region (D and E) are artifacts in frozen section preparation.

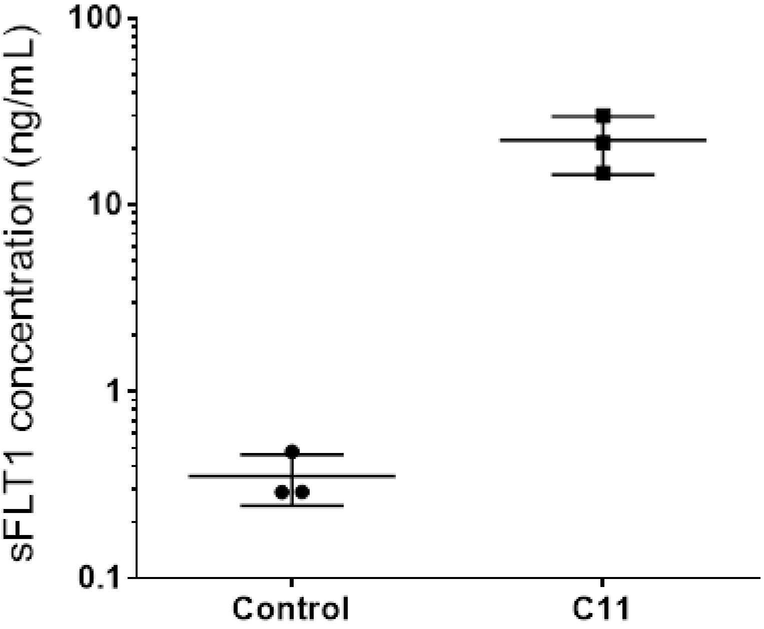

Figure S2. Expression of recombinant protein in pig retinal explants transduced with AAV2.7m8 vector with the original sFlt1-expression cassette under control of CMV early enhancer/promoter and SV40 poly-adenylation signal compared with AAV2.7m8 with codon-optimized Flt1 under control of C11 combination of regulatory elements. Human Flt1 receptor ectodomain sFlt1 was used as a reporter in the optimization screens. Control: AAV2.7m8CMV-sFlt1 (CMV early enhancer/promoter, followed by sFlt1 sequence with SV40 polyA signal), C11: AAV.7m8-C11-CO.sFlt1. Mean values ± SEM are indicated.

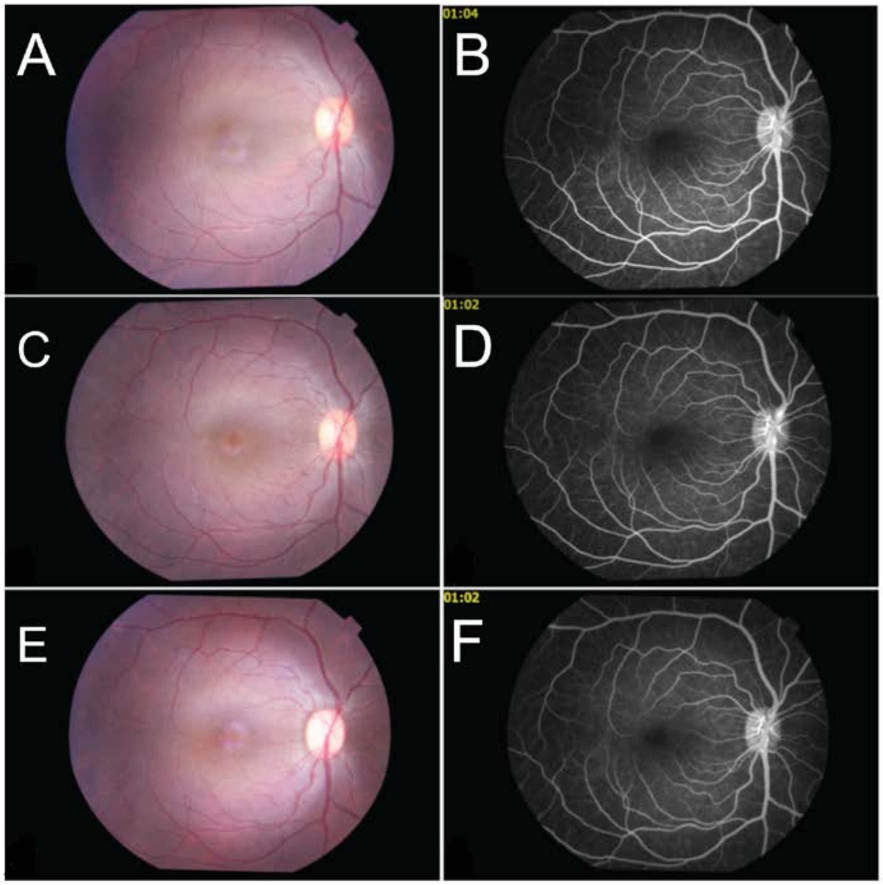

Figure S3: IVT administration of ADVM-022 does not result in changes in retinal morphology, vascular integrity or optic nerve head. Representative color fundus photographs and early phase fluorescence angiograms of eyes receiving ADVM-022 from baseline to 12.5 months. Photographs were obtained from the eye A255 OD at baseline (**A** and **B**), 6 months (**C** and **D**), and 12.5 months (**E** and **F**).

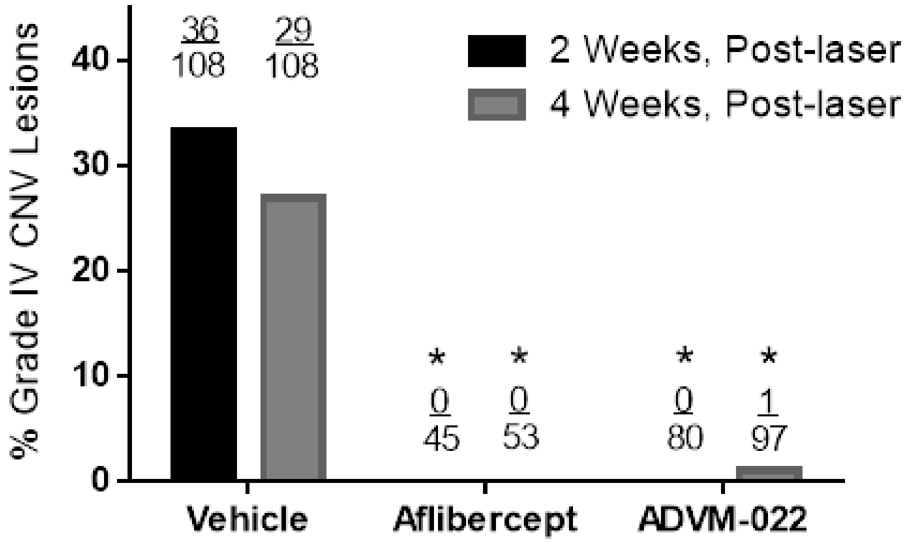

Figure S4. Single dose IVT ADVM-022 significantly reduces the incidence of Grade IV lesions when administered 56 days prior to laser-induced CNV. 6 animals received bilateral IVT injections of ADVM-022 (2×10^{12} vg/eye), and 6 animals of control group received IVT vehicle injections. Laser induction of CNV was performed 56 days after dosing. Standard of care aflibercept was used as a positive control. It was injected immediately after the laser procedure, to the eyes of 3 previously treatment-naïve animals. The Grade IV lesion incidence was scored 2 and 4 days after the CNV induction. The decrease in Grade IV lesions between the ADVM-022 and aflibercept groups compared with the control group was statistically significant ($p < 0.0001$). The incidence of Grade IV lesions was similar between the ADVM-022 and aflibercept groups.

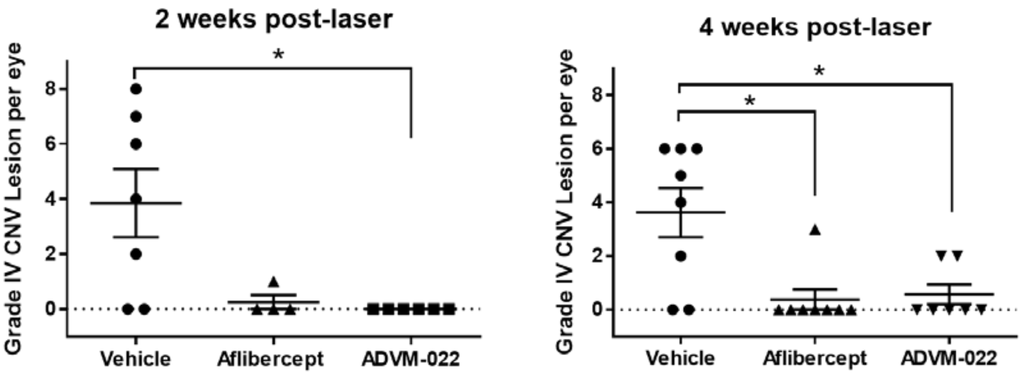

Figure S5. Single dose IVT ADVM-022 reduces incidence of grade IV lesions per individual eye, in the laser-induced CNV African green monkey model, 13 months after the therapy delivery. To address the potential eye-to-eye variability in response to the treatments, the treatment groups were also compared based on the counts of Grade IV lesions per eye, using the Mann-Whitney U-test. This analysis confirmed a statistically significant lower incidence of Grade IV lesions in the eyes treated with ADVM-022 compared with vehicle 2 weeks and 4 weeks post laser. The effect of aflibercept did not reach statistical significance at 2 weeks due to the low number of lesions observable for scoring, and it was statistically significant at 4 weeks post laser. There was no significant difference between ADVM-022 and aflibercept at either time point. At 2 weeks post-laser, 7 vehicle-injected eyes, 4 aflibercept injected eyes and 6 ADVM-022 injected eyes were suitable for scoring; at 4 weeks, 8 vehicle treated eyes, 8 aflibercept-injected eyes and 7 ADVM-022-injected eyes were scored by the treatment-masked investigator. Means with SEM are indicated. * $P < 0.05$.
